# Luminal surface proteome of the brain vasculature uncovers blood-brain barrier regulators

**DOI:** 10.1126/science.aea2100

**Published:** 2026-04-09

**Authors:** Zijian Zhu, Zuzhi Jiang, Yupu Wang, Khanh Nguyen, Yuxiang Zhang, Cameron Genxuan Lian, D. R. Mani, Jun Zheng, Lang Ding, Shihong Max Gao, Alps Xia, Anne Kuszpit, Sarah Lindo, Crystall Lopez, Catherine Lindsey, Brooke Groff, Xinhong Chen, Jiahui Wu, Weiliang Xia, Wei Li, Xiaorong Liu, Viviana Gradinaru, Steven A. Carr, Namrata D. Udeshi, Jiefu Li

**Affiliations:** 1 Janelia Research Campus, Howard Hughes Medical Institute, Ashburn, VA 20147, USA.; 2 Zhiyuan College and School of Biomedical Engineering, Shanghai Jiao Tong University, Shanghai, 200240, China.; 3 Yuanpei College, Peking University, Beijing, 100871, China.; 4 The Broad Institute of MIT and Harvard, Cambridge, MA 02142, USA.; 5 Howard Hughes Medical Institute and Division of Biology & Biological Engineering, California Institute of Technology, Pasadena, CA, 91125, USA.; 6 Retinal Neurophysiology Section, National Eye Institute, Bethesda, MD 20892, USA.; 7 Departments of Biology, Ophthalmology, and Psychology and Program in Fundamental Neuroscience, University of Virginia, Charlottesville, VA 22904, USA.

## Abstract

At the blood-tissue interface, vasculature luminal surface is critical for molecular transport, signaling transduction, and cell extravasation. Here, we present a method for proteomic profiling of the vasculature luminal surface *in vivo*, broadly applicable to any vertebrate. Quantitative mass spectrometry revealed the luminal surface proteome of the mouse brain vasculature and its temporal evolution from development to aging. *In vivo* genetic perturbation found that the arginine transporter SLC7A1 and the nitric oxide synthase NOS3 are needed for blood-brain barrier integrity in neonatal but not adult mice, whereas the hyaluronan degradation enzyme HYAL2 safeguards the barrier throughout the lifespan. By characterizing the proteomic dynamics of the vasculature luminal surface, the study links the metabolism of nitric oxide and hyaluronan to blood-brain barrier integrity.

The evolution of the vasculature system is a critical evolutionary achievement. Before it emerged, organismal size and complexity was bottlenecked by the diffusion limit of oxygen and nutrients. Primitive vasculature systems arose in early bilaterians over 600 million years ago whereas closed circulatory systems with an actively pumping heart and specialized blood vessels appeared in annelids and early vertebrates (1), coinciding with the Cambrian explosion when organismal size and diversity drastically increased in a short evolution period (2, 3). Enabled by the vasculature system, metabolite exchange among distal tissues mitigated the necessity of metabolic self-sufficiency of individual tissue and thus augmented the functional specialization and mutual dependency of organs such as brain, kidney, and liver. The evolution of organs conversely drove the specialization of their innervating blood vessels (Fig. 1A). For instance, the brain vasculature features a tightly sealed and highly selective blood-brain barrier, while kidney and liver host fenestrated and sinusoid capillaries, respectively, to allow free exchanges of molecules and even cells (4, 5).

The luminal surface of the vasculature system is primarily constituted by the apical surface of endothelial cells, the extracellular glycocalyx, and plasma-borne factors. Located at the interface between circulating blood and surrounding tissues, the luminal surface mediates a wide spectrum of vital processes through cell-surface transporters and receptors, including nutrient delivery, waste removal, and endocrine signaling (6). It also serves as a barrier and modulator of immunity, controlling the passage of immune molecules and cells into tissues (6). Its vast size estimated to be near 1,000 m^2^ in humans (7) underscores its physiological importance across the body. Moreover, luminal surface dysfunction and pathology has been implicated in many life-threatening diseases including sepsis, thrombosis, atherosclerosis, hypertension, and tumor metastasis (8–12). To decode the diverse physiological functions of the vasculature luminal surface at a molecular level and unravel its mechanistic roles in disease pathology, it is essential to develop a comprehensive map of its protein composition and dynamics.

Here, we present a generalizable method for profiling the vasculature luminal surface proteome, which does not require genetic access and is potentially broadly applicable to any vertebrate. Combining quantitative proteomics and *in vivo* genome editing, we revealed how the brain vasculature luminal surface changes across the lifespan and uncovered age-specific regulators of blood-brain barrier integrity.

## *In vivo* peroxidase-mediated protein labeling at the vasculature luminal surface

**Fig. 1.**
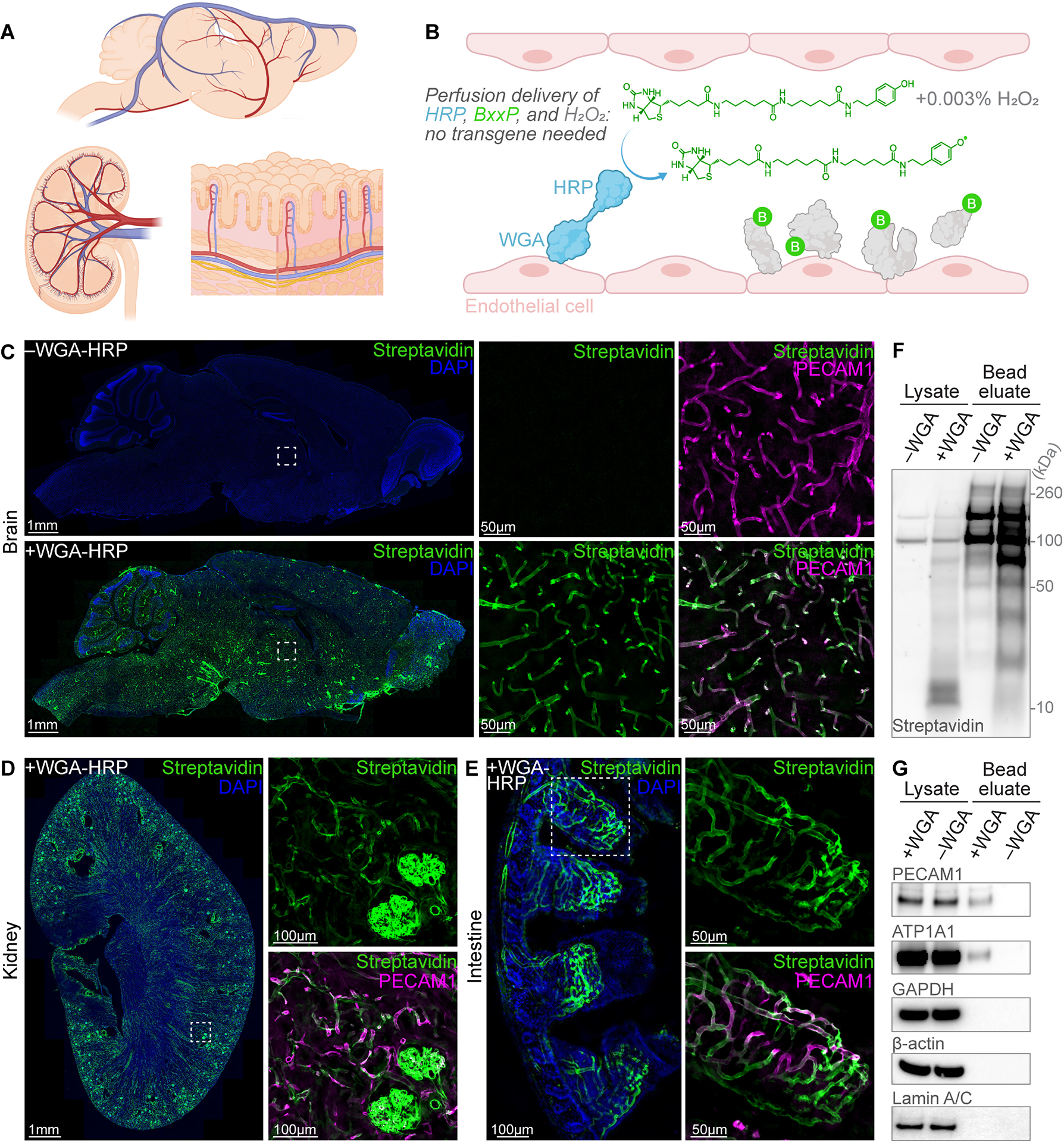
*In vivo* biotinylation of vasculature luminal surface proteins. (**A**) Illustration of blood vessels in various organs: brain, kidney, and intestine. (**B**) Schematic of the method. Wheat germ agglutinin (WGA) anchors the horseradish peroxidase (HRP) to the vasculature luminal surface. HRP converts biotin-xx-phenol (chemical structure in green) to phenoxyl radicals (13), which covalently biotinylate nearby proteins (“B” dots in green). (**C to E**) Streptavidin and anti-PECAM1 staining of brain (C), kidney (D), and intestine (E). Boxed regions are enlarged on the right. DAPI, nucleus staining. We note that biotinylation may partially mask PECAM1 epitopes, resulting in reduced PECAM1 staining in Streptavidin-high regions. (**F**) Streptavidin blot of raw brain lysates and post-enrichment bead eluates. (**G**) Western blots of endothelial cell marker PECAM1 and cell-surface Na^+^/K^+^ ATPase ATP1A1, as well as intracellular proteins GAPDH, β-actin, and lamin A/C, in raw brain lysates and post-enrichment bead eluates. −WGA, WGA-HRP omitted. +WGA, WGA-HRP perfused.

Peroxidases, particularly the horseradish peroxidase (HRP), are robust tools for catalyzing proximity labeling reactions at the cell surface (13–16). Genetically-encoded HRP transgenes (14, 15) enable *in situ* cell-surface proteomics in intact animal tissues but are only applicable to genetically-tractable model organisms such as the fruit fly and mouse. To eliminate the requirement of genetic access, we used wheat germ agglutinin (WGA), a lectin that binds specifically to N-acetylglucosamine and sialic acid residues on glycoproteins and glycolipids, to anchor HRP to the vasculature luminal surface by cardiac perfusion of WGA-HRP conjugated proteins (Fig. 1B). Luminal surface-localized HRP can then catalyze the biotinylation of nearby proteins using the membrane-impermeable substrate biotin-xx-phenol and hydrogen peroxide (13, 14) delivered via perfusion (Fig. 1B).

As shown in the mouse brain (Fig. 1C), this *in vivo* biotinylation reaction was WGA-HRP-dependent, exhibiting little background when WGA-HRP was omitted (Fig. 1C, up row). Across all brain regions, the biotin signal was specific to the vasculature and colocalized with the blood vessel marker PECAM1 (Fig. 1C, bottom row). Likewise, this approach also biotinylated the vasculature luminal surface of kidney, covering cortical, glomerular, and medullary blood vessels (Fig. 1D) and intestine, including the capillary network of intestinal villi (Fig. 1E), as well as muscle and liver (fig. S1, A and B). To demonstrate the broad applicability of this method to diverse organisms, we tested it on the northern treeshrew (*Tupaia belangeri*; fig. S2A), a species evolutionarily close to primates (17) (fig. S2B). Streptavidin staining showed extensive, WGA-HRP-dependent biotinylation in the brain and muscle vasculature (fig. S2, C and D).

**Fig. 2.**
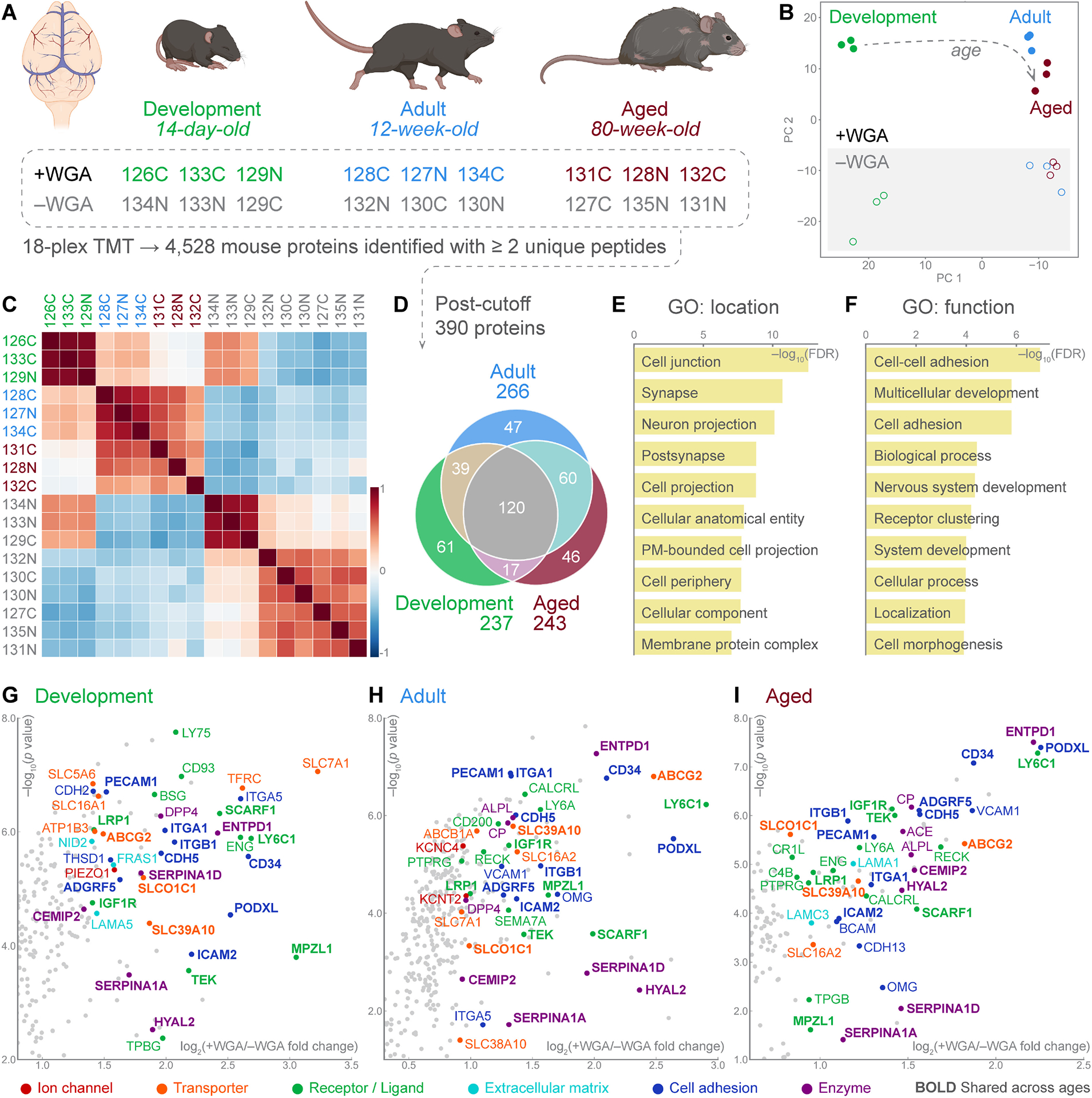
Luminal surface proteome of mouse brain vasculature across the lifespan. (**A**) Design and TMT tag usage of the 18-plex quantitative proteomic experiment. Each age comprises three biotinylated replicates (colored TMT tags: green, development; blue, adult; red, aged) and three non-biotinylated controls (gray TMT tags). (**B**) Principal component analysis of the proteome. Solid dots, +WGA, biotinylated. Empty dots, −WGA, control. (**C**) Correlation of 18 TMT channels. (**D**) Venn diagram of the post-cutoff proteome. (**E and F**) Top ten gene ontology features of the post-cutoff 390 proteins: location (E) and function (F). FDR, false discovery rate. (**G to I**) Most enriched proteins at the development (G), adult (H), and aged (I) stages. Proteins are colored based on their molecular families: red, ion channel; orange, transporter; green, receptor or ligand; cyan, extracellular matrix; blue, adhesion molecule; purple, enzyme. Bold font highlights shared proteins among three panels. +WGA, WGA-HRP perfused. −WGA, WGA-HRP omitted.

**Fig. 3.**
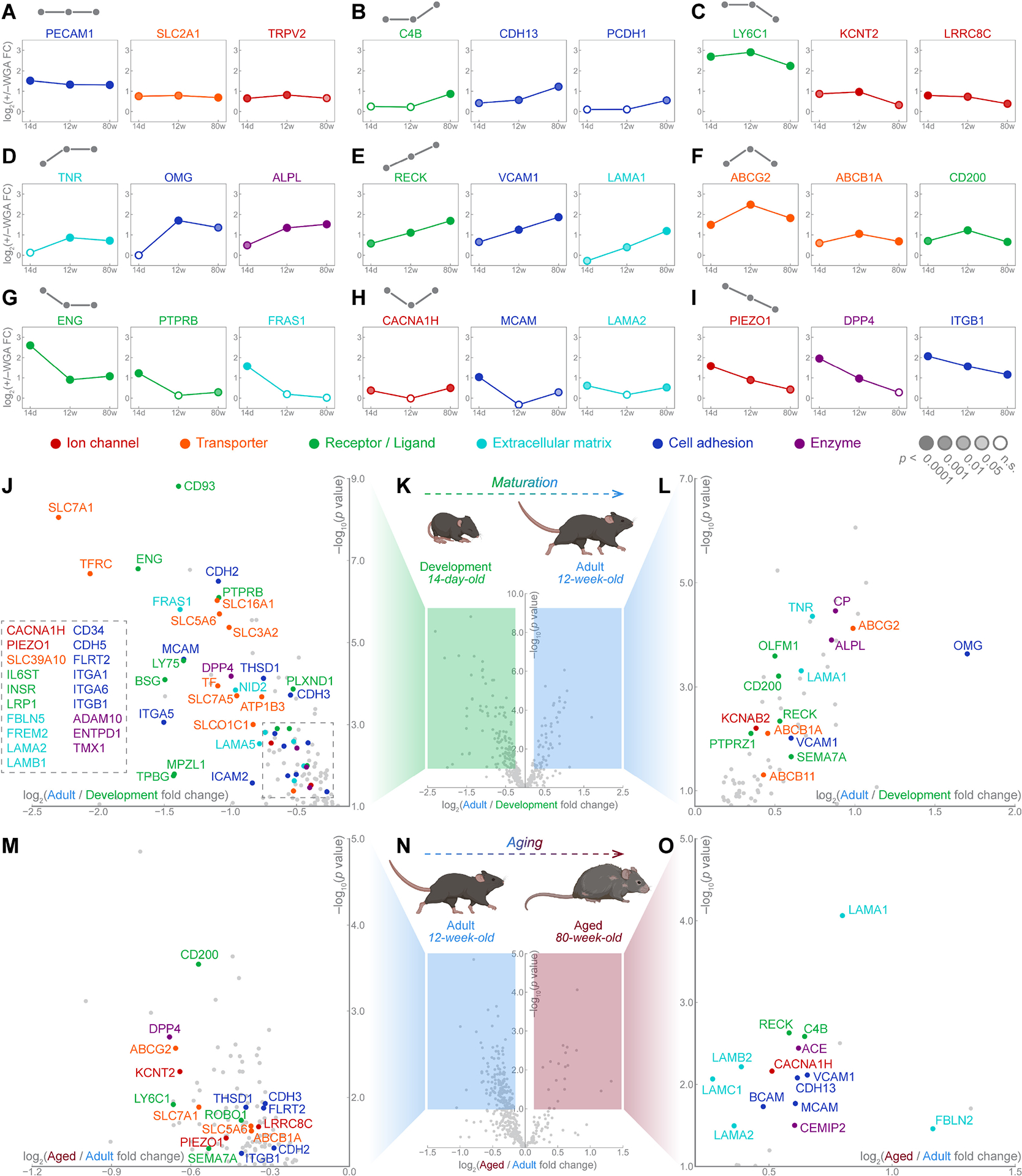
Proteomic dynamics from development through adulthood to aging. (**A to I**) Various dynamic patterns, such as static (A), unidirectionally rising (E), and unidirectionally reducing (I), and their example proteins. Y axis, log_2_(+WGA/–WGA fold change). +WGA, WGA-HRP perfused. −WGA, WGA-HRP omitted. FC, fold change. 14d, 14-day-old. 12w, 12-week-old. 80w, 80-week-old. Error bar, standard deviation. Asterisk, *p* value associated with the +WGA/–WGA fold change to assess whether a protein is enriched at each stage. ***, *p* < 0.001. **, *p* < 0.01. *, *p* < 0.05. n.s., not significant. (**J to L**) Down- (J) and up- (L) regulated proteins in the development-to-adult transition (K). (**M to O**) Down- (M) and up- (O) regulated proteins in the process of aging (N). Proteins are colored based on their molecular families: red, ion channel; orange, transporter; green, receptor or ligand; cyan, extracellular matrix; blue, adhesion molecule; purple, enzyme.

Biochemical assays on the mouse brain samples indicated that this method biotinylated and enriched proteins with a wide range of molecular weights (Fig. 1F; fig. S3A), including the endothelial cell marker PECAM1 and the essential cell-surface Na^+^/K^+^ ATPase ATP1A1 (Fig. 1G, upper two rows) but not abundant intracellular proteins such as GAPDH, β-actin, or lamin A/C (Fig. 1G, bottom three rows), validating the cell-surface specificity of this method. We further performed streptavidin blots, sliver stains, and Western blots on the kidney (fig. S3, B to D) and intestine (fig. S3, E to G) samples, along with quantitative proteomic analyses of both brain (Fig. 2 and 3; table S1 to S4) and kidney (fig. S4; table S5 and S6), collectively demonstrating the broad applicability of this method across diverse organs.

We note that the primary amine reactive chemical Sulfo-NHS-biotin and its various derivatives (fig. S5A), which are commonly used for testing vasculature permeability and were proposed for proteomic profiling of the vasculature luminal surface (18), are not suitable for the latter purpose due to the lack of compartment specificity in almost all organs. In the brain, it leaked into cerebrospinal fluid and thus labelled the pia mater and many non-vasculature structures (fig. S5B). In the kidney, it passed through glomerular filtration and therefore labelled all urine-collecting compartments such as the major calyx (fig. S5C). In the intestine, the epithelium and other nearby structures were also biotinylated (fig. S5D).

## Luminal surface proteome of the mouse brain vasculature

Blood vessels in the brain are structurally and functionally specialized, featuring its tightly sealed and regulated blood-brain barrier for selective transportation, immune privilege, and neurovascular coupling (4, 19–22). With salient clinical implications for drug delivery and neuroimmunology, the development and maintenance of the blood-brain barrier have been long-studied yet remain incompletely understood. Furthermore, it is largely unknown how brain blood vessels change in aging and how vasculature aging impacts brain aging. To provide a quantitative, holistic view of the blood-brain interface, here we profiled the luminal surface proteome of the brain vasculature in developing (postnatal day 14), adult, and aged mice (Fig. 2A).

Streptavidin staining showed that the WGA-HRP and chemical perfusion method (Fig. 1B) produced brain-wide vasculature biotinylation in both postnatal day 14 (fig. S6A) and 80 weeks old (fig. S6B) mice. To better quantify the proteomic dynamics from development to aging, we used 18-plex tandem mass tag (TMT)-based mass spectrometry (23) (Fig. 2A): each age group contained three biotinylated replicates (+WGA, colored TMT tags in Fig. 2A) and three non-biotinylated controls to capture the background (–WGA, gray TMT tags). Streptavidin blotting of raw brain lysates and post-enrichment bead eluates showed highly comparable labeling and enrichment among biological replicates across ages (fig. S6, C to E). Peptides of enriched proteins were released from streptavidin beads by on-bead tryptic digestion, followed by TMT multiplexing, fractionation, and liquid chromatography-tandem mass spectrometry analysis (LC-MS/MS) (fig. S6F).

From this 18-plex TMT experiment, a total of 4,528 mouse proteins were detected with 2 or more unique peptides (Fig. 2A; table S1), exhibiting high correlations among biological replicates (Fig. 2C). Principal component analysis also revealed that replicates of each group clustered respectively, with the top two principal components corresponding to the age and the experimental condition (+WGA vs. −WGA) (Fig. 2B). The age trajectory in principal component analysis (gray line, Fig. 2B), as well as the correlations (Fig. 2C), revealed that the developmental brain vasculature possessed a luminal surface milieu distinct from the adult and aged. Compared with the profound development-to-adult transition, the proteome remained largely stable during aging.

To filter out endogenously biotinylated proteins, non-specific bead binders, and other contaminants, we performed cutoff analysis based on the biotinylated-to-control (‘+WGA’ to ‘–WGA’) ratios as previously described (13–15, 24, 25). Shown in fig. S6, G to I, proteins of each age were ranked descendingly by the biotinylated-to-control ratio. Their accumulative true positive rates and false positive rates were calculated based on UniProt references, with a cutoff position set where the value of ‘true positive rate – false positive rate’ was maximal. Proteins ranked above this cutoff position were retained: 237, 266, and 243 proteins for development, adult, and aged, respectively, totaling 390 proteins with substantial overlap across ages (Fig. 2D; table S2; details in the Materials and Methods). Their gene ontology featured cell-surface localization (Fig. 2E) and diverse cellular functions such as cell adhesion, development, and receptor signaling (Fig. 2F). We also performed cutoff analysis using an alternative approach by setting a defined biotinylated-to-control ratio and retaining all statistically significant proteins above this ratio (fig. S6, K to M) and obtained a mostly overlapping but slightly larger proteome (fig. S6, J and N; table S3) with similar features (fig. S6O).

## Proteome dynamics across the lifespan

Fig. 2, G to I highlighted the most enriched proteins at the developmental, adult, and aged stages, respectively, and showed that the proteome comprised all cell-surface protein categories: ion channels (red), transporters (orange), receptors and ligands (green), extracellular matrix components (cyan), adhesion molecules (blue), and enzymes (purple). Classic blood vessel markers, such as CD34, PECAM1 (CD31), TEK (TIE2), and CDH5 (VE-cadherin), were constantly abundant across the lifespan (bold, Fig. 2, G to I). Additionally, integrins ITGA1 and ITGB1, adhesion GPCR ADGRF5, scavenger receptors SCARF1 and LRP1, zinc transporter SLC39A10 (ZIP10), thyroid hormone transporter SLCO1C1, hyaluronidases HYAL2 and CEMIP2, protease inhibitors SERPINA1s, and many other proteins from diverse functional families were also highly enriched at all stages (bold, Fig. 2, G to I), exemplifying the shared luminal surface proteome across ages (Fig. 2D; table S2E).

Whereas the blood vessel marker PECAM1 and the housekeeping glucose transporter SLC2A1 (GLUT1) were expressed stably throughout the lifespan (Fig. 3A), most proteins at the luminal surface exhibited various dynamic patterns in the transition from development through adulthood into aging (Fig. 3, B to I; table S4). Proteins belonging to the same molecular families or sharing similar functions (category-colored tiles in Fig. 3, A to I) often exhibited distinct and sometimes opposite temporal dynamics. For instance, each member of the laminin family—critical elements of the extracellular matrix—had a unique temporal pattern (fig. S7E), suggesting functional specification of laminin subunits in vasculature development, maintenance, and aging. The same complexity was also observed for integrins, cadherins, and potassium channels (fig. S7, A and F).

Aligned with the principal component and correlation analyses (Fig. 2, B and C), the development-to-adult transition (Fig. 3K) involved a global remodeling of the blood vessel luminal surface, including a collective down-regulation of angiogenesis factors such as ENG (26, 27), CDH5 (28–30), PTPRB (31), PLXND1 (32), and PIEZO1 (33, 34) (Fig. 3J), as well as many adhesion molecules mediating cell-cell or cell-extracellular matrix interactions (blue, Fig. 3J). Alongside angiogenesis factors, solute carrier transporters (orange, Fig. 3J; fig. S7B) were tuned down together while ATP-binding cassette transporters (orange, Fig. 3L) were collectively elevated, likely reflecting a metabolic transition from the developmental need of amino acids and monocarboxylates to the clearance of xenobiotics and metabolites since ABCB1 and ABCG2 are essential efflux transporters required for molecular exportation at the blood-brain barrier (35–38).

In contrast to the developmental dynamics, both ATP-binding cassette and solute carrier transporters dropped down in aging (fig. S7B), including efflux transporters ABCB1 and ABCG2, amino acid transporter SLC7A1, and vitamin transporter SLC5A6 (orange, Fig. 3M), suggesting compromised waste removal and nutrient uptake in aging. Besides the declining of metabolic exchange machineries, aging also created a pro-inflammatory luminal milieu with reduced CD200 (OX-2), an immuno-suppressive signal (39–41) (Fig. 3M), and elevated VCAM1 and MCAM (Fig. 3O), two inflammation-inducible adhesion molecules mediating leukocyte extravasation (42–45), in addition to a core member of the complement pathway C4B (46) (Fig. 3O). Moreover, the mechanical environment and adaptability was altered in aging from several different but synergistic aspects: 1) Concerted elevation of extracellular matrix components (cyan, Fig. 3O), especially several laminin subunits, implied a stiffer and less stretchable environment. 2) PIEZO1, a force-gated ion channel for blood flow sensation and adaptation (33, 34, 47), and LRRC8C, a subunit of the volume-regulated anion channel for osmolarity adaptation (48–50) (red, Fig. 3M), as well as several potassium channels (fig. S7A), showed reduced expression amounts, undermining the adaptability of endothelial cells. 3) ACE, the angiotensin-converting enzyme increasing blood pressure (51, 52), and CACNA1H, a voltage-gated calcium channel subunit whose gain-of-function mutation causes hypertension (53), were elevated in aging (Fig. 3O). Together with the pro-inflammatory immune status, these aging alterations could create higher vascular rigidity and lower capacity for physiological adaptation, potentially contributing to hypertension and other age-associated vascular malfunction.

## Neonates require SLC7A1 for blood-brain barrier integrity

**Fig. 4.**
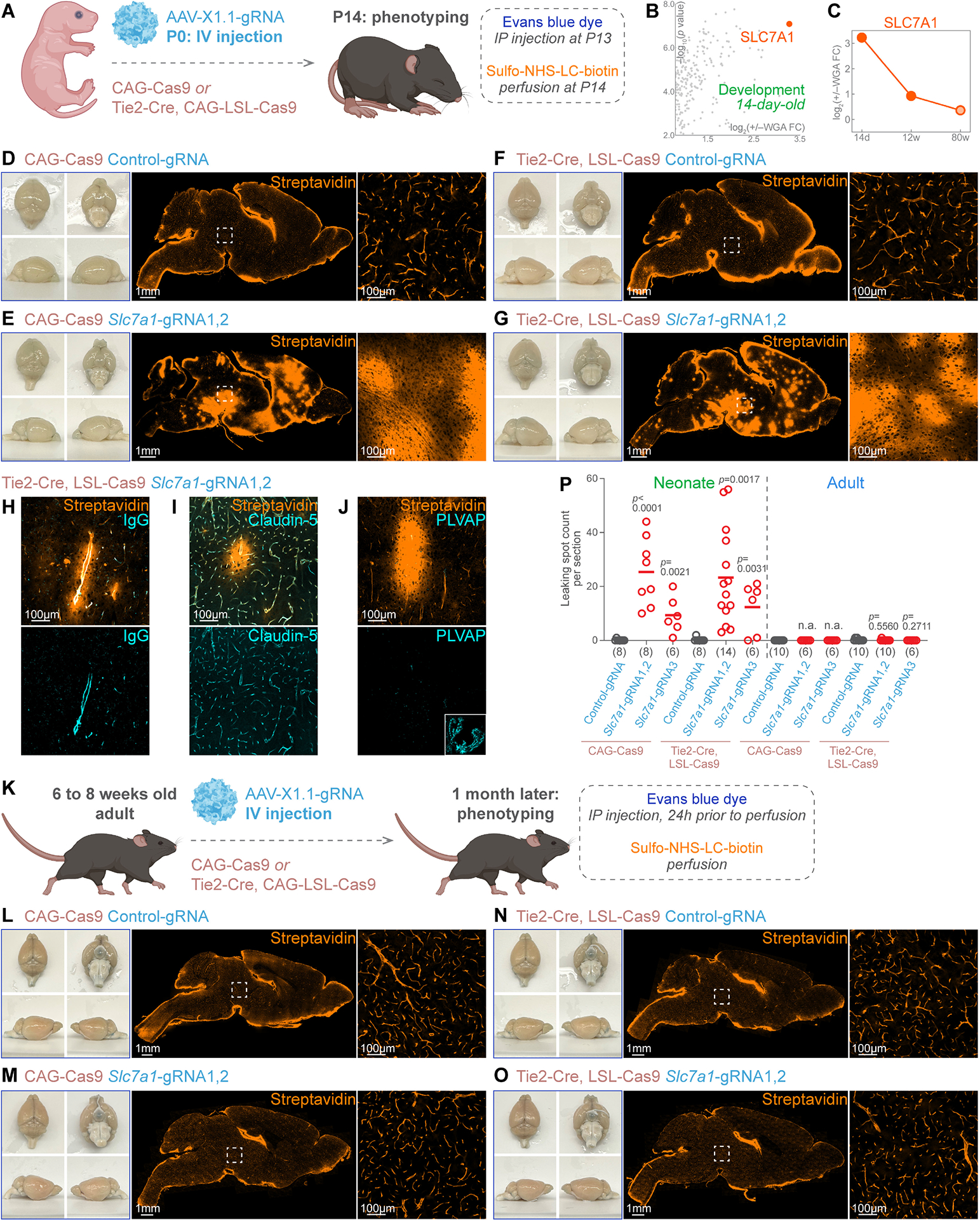
Neonatal but not adult blood-brain barrier requires SLC7A1. (**A**) Schematic of the viral-genetic strategy for knocking out genes in brain endothelial cells in neonatal mice. Adeno-associated virus (AAV) with the X1.1 capsid (55), which encodes one or two guide RNAs (gRNAs), is injected intravenously (IV) into postnatal day 0 (P0) mice that express body-wide Cas9 (CAG-Cas9) (56) or endothelial cell specific Cas9 (Tie2-Cre (57), CAG-LSL-Cas9 (56)). The albumin-binding dye Evans blue is injected intraperitoneally (IP) on postnatal day 13 (P13) and examined on postnatal day 14 (P14) after cardiac perfusion of another barrier permeability tracer, Sulfo-NHS-LC-biotin. (**B**) Enrichment of SLC7A1 in the proteome at postnatal day 14. +WGA, WGA-HRP perfused. −WGA, WGA-HRP omitted. FC, fold change. (**C**) Expression dynamics of SLC7A1. Y axis, log_2_(+WGA/–WGA fold change). +WGA, WGA-HRP perfused. −WGA, WGA-HRP omitted. FC, fold change. 14d, 14-day-old. 12w, 12-week-old. 80w, 80-week-old. Error bar, standard deviation. Asterisk, *p* value associated with the +WGA/–WGA fold change to assess whether SLC7A1 is enriched at each stage. ***, *p* < 0.001. **, *p* < 0.01. *, *p* < 0.05. n.s., not significant. (**D and F**) Control guide RNAs in body-wide (D) or endothelial cell specific (F) Cas9 mice at the neonatal stage. Left, whole brain photos showing the Evans blue stain. Middle, streptavidin staining of the brain sagittal section for Sulfo-NHS-LC-biotin detection. Right, zoom-in of the boxed region. (**E and G**) *Slc7a1* knockout by two guide RNAs (*Slc7a1*-gRNA1,2) in body-wide (E) or endothelial cell specific (G) Cas9 mice at the neonatal stage. (**H to J**) Immunostaining of mouse IgG (H), Claudin-5 (I), and PLVAP (J). Inset in (J) shows the choroid plexus, where blood vessels express PLVAP. (**K**) Schematic of the viral-genetic strategy for knocking out genes in brain endothelial cells in young adult mice. AAV-X1.1 encoding one or two guide RNAs is injected intravenously into 6 to 8 weeks old mice expressing Cas9. One month after AAV injection, Evans blue is injected intraperitoneally and examined next day after cardiac perfusion of Sulfo-NHS-LC-biotin. (**L and N**) Control guide RNAs in body-wide (L) or endothelial cell specific (N) Cas9 mice in adulthood. (**M and O**) *Slc7a1* knockout by two guide RNAs (*Slc7a1*-gRNA1,2) in young adult mice. M, body-wide Cas9. O, endothelial cell specific Cas9. (**P**) Quantification of Sulfo-NHS-LC-biotin leaking spots in control and *Slc7a1* knockout. The total number of mice used for each experimental condition, from left to right, was: 4, 4, 3, 4, 7, 3, 5, 3, 3, 5, 5, and 3. From each mouse, two sagittal brain sections were stained with streptavidin, imaged, and quantified. Numbers in parentheses indicate the total number of brain sections quantified for each condition. Two-tailed *t* test was used to compare each knockout with its corresponding control. n.a., not applicable because a *p* value cannot be calculated when two groups have identical values.

Solute carrier transporters transport vital nutrients, such as amino acids, lipids, monocarboxylates, and vitamins (54). Collective enrichment of solute carrier transporters in neonates (orange, Fig. 3J; fig. S8A) suggested their functional relevance in developing brains. To test whether loss of any of them causes nutrient deficiency and brain development defects such as microcephaly, we devised a CRISPR-based viral-genetic strategy to knock out select genes in brain endothelial cells in neonatal mice (Fig. 4A): a brain endothelial cell specific adeno-associated virus (AAV), AAV-X1.1 (55), was used to deliver one or two guide RNAs by intravenous injection into newborn mice that express body-wide Cas9 (56). None of the four tested solute carrier transporters or the transferrin receptor TFRC (fig. S8) caused noticeable defects of brain size, brain shape, or body weight when knocked out individually (for instance, no body weight reduction in *Slc7a1* knockout; fig. S9G), prompting us to examine their potential non-metabolic functions in the brain vasculature.

Loss of the amino acid transporter SLC7A1 caused severe blood-brain barrier leakage, which was not observed in the loss of the other three transporters or the transferrin receptor. Two weeks after AAV injection, the albumin-binding dye Evans blue and the primary amine reactive chemical Sulfo-NHS-LC-biotin (fig. S5A) were used to probe blood-brain barrier permeability by intraperitoneal injection and cardiac perfusion, respectively (Fig. 4A). Compared with the control (Fig. 4D), the *Slc7a1* knockout brain was slightly blue-tinted (left, Fig. 4E) and showed intense streptavidin staining across many brain regions (middle and right, Fig. 4E; quantified in Fig. 4P), indicating the leakage of Evans blue-bound albumin and Sulfo-NHS-LC-biotin into brain parenchyma. None of the antibodies we tested provided specific immunostaining for SLC7A1. Therefore, we knocked out *Slc7a1* using another independent, single guide RNA (gRNA3, fig. S9B) and observed the same leaking phenotypes (fig. S9C and Fig. 4P), excluding the possibility of guide RNA off-targeting. Like most other AAV capsids, AAV-X1.1 also transduces liver hepatocytes (55). To further restrict the knockout to brain endothelial cells, we used Tie2-Cre (57) and loxP-gated Cas9 (56) to only express Cas9 in endothelial cells and observed identical phenotypes as body-wide Cas9, using either dual or single guide RNAs (Fig. 4, F and G and fig. S9D; quantified in Fig. 4P), ruling out the possibility that the leaking phenotype was a secondary effect caused by SLC7A1 loss in hepatocytes.

In *Slc7a1* knockout experiments, the leakage of Evans blue-bound albumin indicated increased permeability to macromolecules, which was further supported by the accumulation of endogenous IgG at the leaking sites (Fig. 4H). This prompted us to investigate whether the loss of SLC7A1 affected tight junction integrity. The leaking sites did not exhibit a reduction in Claudin-5 expression (Fig. 4I), nor did they show ectopic expression of PLVAP, a marker of fenestrated blood vessels (Fig. 4J). These findings suggest that SLC7A1 loss compromises the blood-brain barrier independently of tight junction disruption.

SLC7A1 was the most enriched luminal surface protein at the developmental stage (Fig. 4B) and was down-regulated after maturation (Fig. 4C). Human RNA-sequencing found that *SLC7A1* is specific to endothelial cells in the brain and is down-regulated in adulthood (58) (fig. S9A), exhibiting the same dynamics as its mouse ortholog (Fig. 4C). We then asked whether the blood-brain barrier in adulthood still requires SLC7A1 for its integrity. To answer this, we used the same viral-genetic strategy (Fig. 4K) by intravenously injecting guide RNA AAVs into adult mice expressing body-wide or endothelial cell specific Cas9 and, after one month, examining blood-brain barrier permeability by Evans blue and Sulfo-NHS-LC-biotin. In contrast to the severe leakage observed in neonates (Fig. 4, E and G and fig. S9, C and D), *Slc7a1* knockout in adult mice did not cause any leakage (Fig. 4, M and O and fig. S9, E and F), showing identical barrier integrity as the control (Fig. 4, L and N; quantified in Fig. 4P). Thus, SLC7A1 is a neonate-specific regulator of the blood-brain barrier and is dispensable in adulthood, consistent with its higher expression in neonates than in adults (Fig. 4, B and C).

## Neonatal blood-brain barrier requires nitric oxide synthesis

**Fig. 5.**
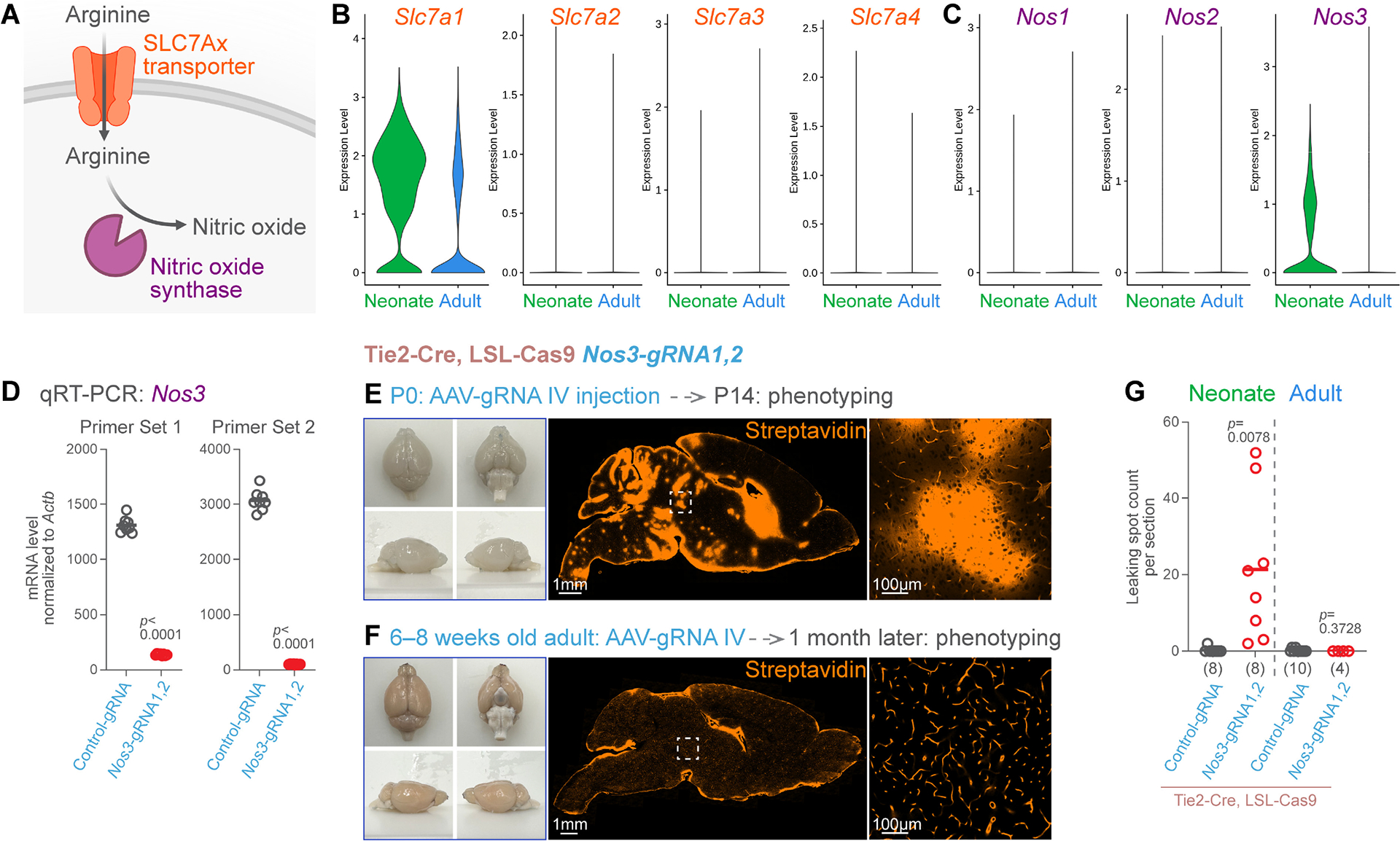
Nitric oxide synthase NOS3 is required at the neonatal blood–brain barrier but not in adults. (**A**) Diagram of the nitric oxide synthesis pathway. Solute carrier transporters SLC7A1–SLC7A4 mediate the intracellular uptake of the cationic amino acid arginine, which is subsequently converted into nitric oxide by nitric oxide synthases (NOS1–NOS3). (**B and C**) mRNA expression of *Slc7a1*–*Slc7a4* (B) and *Nos1*–*Nos3* (C) in mouse brain endothelial cells, as revealed by single-cell RNA sequencing (61). Neonate, postnatal day 10. Adult, 7 to 11 weeks old. (**D**) Quantitative reverse transcription PCR (qRT-PCR) validation of CRISPR/Cas9-mediated *Nos3* knockout. Two distinct primer pairs (sequences provided in the Materials and Methods) were used to quantify *Nos3* transcript amounts in RNA extracted from two mouse brains, with four PCR technical replicates per brain sample. mRNA expression was normalized to *Actb* (β-actin). Two-tailed *t* test was used to compare each knockout with its corresponding control. (**E and F**) *Nos3* knockout by two guide RNAs (*Nos3*-gRNA1,2) in endothelial cell specific Cas9 mice at the neonatal stage (E) or in adulthood (F). Left, whole brain photos showing the Evans blue stain. Middle, streptavidin staining of the brain sagittal section for Sulfo-NHS-LC-biotin detection. Right, zoom-in of the boxed region. (**G**) Quantification of Sulfo-NHS-LC-biotin leaking spots in control and *Nos3* knockout. The total number of mice used for each experimental condition, from left to right, was: 4, 4, 5, and 2. From each mouse, two sagittal brain sections were stained with streptavidin, imaged, and quantified. Numbers in parentheses indicate the total number of brain sections quantified for each condition. Two-tailed *t* test was used to compare each knockout with its corresponding control.

As a member of the cationic amino acid transporter family, SLC7A1 plays a critical role in the nitric oxide synthesis pathway (59, 60) by mediating the intracellular uptake of arginine, the substrate used by nitric oxide synthases to generate nitric oxide (Fig. 5A). RNA sequencing (61) revealed that *Slc7a1* is the predominantly expressed one among its family members (*Slc7a1*–*Slc7a4*) in mouse brain endothelial cells and is expressed at a higher amount in neonates compared with adults (Fig. 5B), consistent with our proteomic data (Fig. 4C). Brain endothelial cells express one of the three nitric oxide synthases, *Nos3* (also known as *eNos*), which shows similar elevated expression in neonates relative to adults (Fig. 5C). The parallel developmental expression patterns of *Slc7a1* and *Nos3*, together with their functional relationship within the same biochemical pathway (Fig. 5A), raised the question of whether SLC7A1 is required for the blood–brain barrier through its role in nitric oxide synthesis.

We therefore designed guide RNAs to knock out *Nos3* (Fig. 5D) and found that *Nos3* deletion at the neonatal stage phenocopied *Slc7a1* knockout, resulting in brain-wide Evans blue stain and Sulfo-NHS-LC-biotin leakage (Fig. 5E; quantified in Fig. 5G). *Nos3* knockout in adulthood did not disrupt the blood–brain barrier (Fig. 5F; quantified in Fig. 5G), again recapitulating the phenotype of *Slc7a1* loss. Together, these findings suggest that the integrity of the neonatal, but not adult, blood–brain barrier depends on nitric oxide synthesis, which relies on sequential SLC7A1-mediated arginine transport and NOS3-catalyzed synthesis.

## HYAL2 controls blood-brain barrier integrity across the lifespan

Inspired by the unexpected discovery of SLC7A1 and its downstream nitric oxide synthase NOS3 as blood-brain barrier regulators, we wondered whether the proteome could inform the identification of previously unknown factors for blood-brain barrier maintenance in adulthood and aging, and thus carried out an *in vivo* genetic screen targeting functionally unstudied proteins that were highly enriched in adulthood (fig. S10), using the viral-genetic strategy illustrated in Fig. 4K. None of the knockouts resulted in mortality, abnormal behavior, bleeding, or overt vascular defects. Additionally, blood-brain barrier breakdown was not observed in the loss of PROM1, MPZL1, SCARF1, or SERPINA1D.

**Fig. 6.**
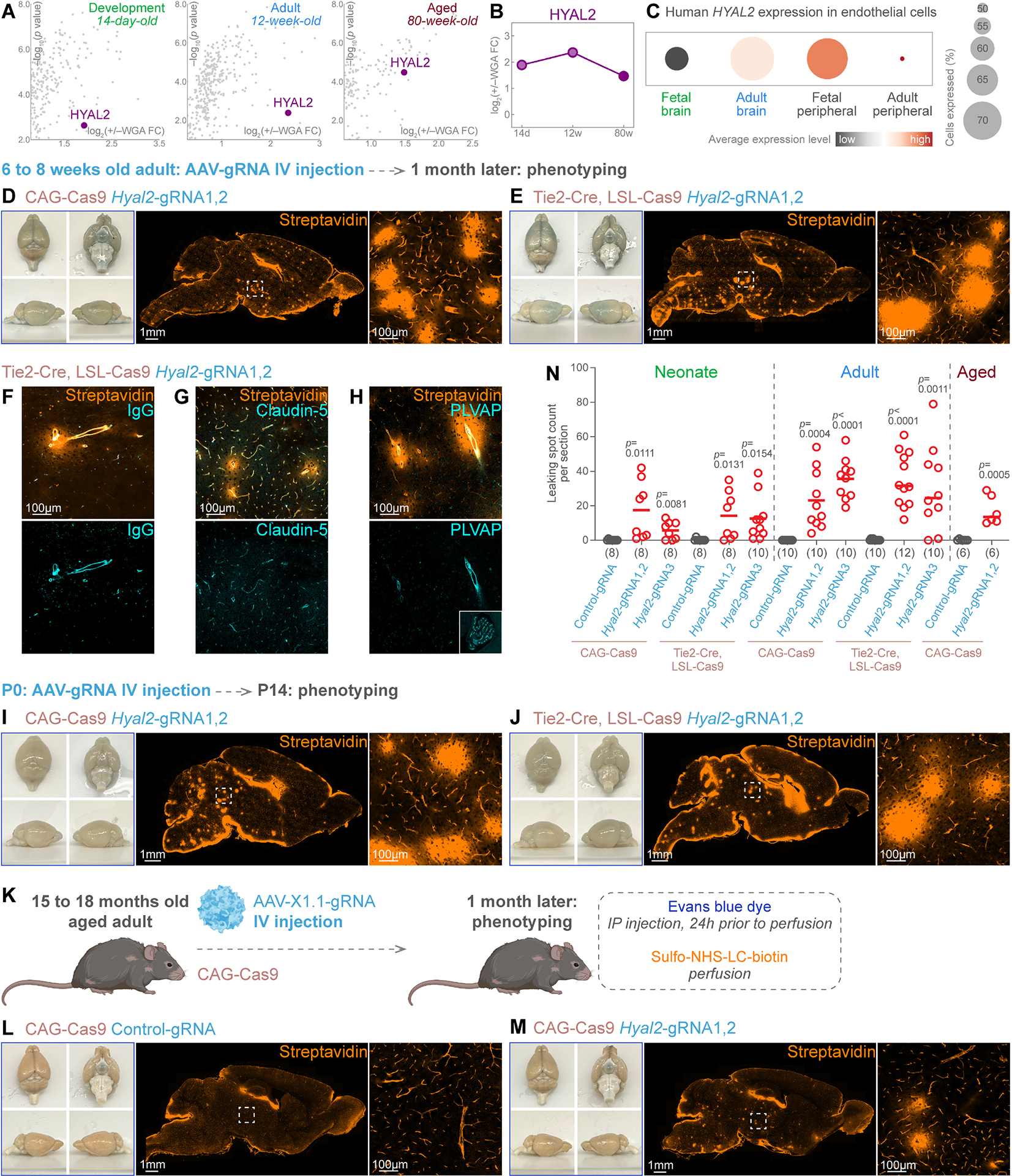
HYAL2 secures blood-brain barrier integrity throughout the lifespan. (**A**) Enrichment of HYAL2 in the proteome across ages. +WGA, WGA-HRP perfused. −WGA, WGA-HRP omitted. FC, fold change. (**B**) Expression dynamics of HYAL2. Y axis, log_2_(+WGA/–WGA fold change). +WGA, WGA-HRP perfused. −WGA, WGA-HRP omitted. FC, fold change. 14d, 14-day-old. 12w, 12-week-old. 80w, 80-week-old. Error bar, standard deviation. Asterisk, *p* value associated with the +WGA/–WGA fold change to assess whether HYAL2 is enriched at each stage. ***, *p* < 0.001. **, *p* < 0.01. *, *p* < 0.05. n.s., not significant. (**C**) *HYAL2* expression in human endothelial cells, revealed by RNA sequencing (58). (**D and E**) *Hyal2* knockout by two guide RNAs (*Hyal2*-gRNA1,2) in body-wide (D) or endothelial cell specific (E) Cas9 mice in adulthood. Left, whole brain photos showing the Evans blue stain. Middle, streptavidin staining of the brain sagittal section for Sulfo-NHS-LC-biotin detection. Right, zoom-in of the boxed region. (**F to H**) Immunostaining of mouse IgG (F), Claudin-5 (G), and PLVAP (H). Inset in (H) shows the choroid plexus, where blood vessels express PLVAP. (**I and J**) *Hyal2* knockout by two guide RNAs (*Hyal2*-gRNA1,2) in body-wide (I) or endothelial cell specific (J) Cas9 mice at the neonatal stage. (**K**) Schematic of the viral-genetic strategy for knocking out genes in brain endothelial cells in aged mice. AAV-X1.1 encoding two guide RNAs is injected intravenously into 15 to 18 months old mice that express body-wide Cas9. One month after AAV injection, Evans blue is injected intraperitoneally and examined next day after cardiac perfusion of Sulfo-NHS-LC-biotin. (**L and M**) Control guide RNAs (L) and *Hyal2*-gRNA1,2 (M) in aged mice that express body-wide Cas9. (**N**) Quantification of Sulfo-NHS-LC-biotin leaking spots in control and *Hyal2* knockout. The total number of mice used for each experimental condition, from left to right, was: 4, 4, 4, 4, 4, 5, 5, 5, 5, 5, 6, 5, 3, and 3. From each mouse, two sagittal brain sections were stained with streptavidin, imaged, and quantified. Numbers in parentheses indicate the total number of brain sections quantified for each condition. Two-tailed *t* test was used to compare each knockout with its corresponding control.

Loss of a hyaluronic acid hydrolase HYAL2, one of the most enriched luminal surface proteins across all ages (Fig. 6, A and B), led to intense Evans blue stain of the brain, particularly the cortex (left, Fig. 6D). Sulfo-NHS-LC-biotin tracing also showed vast leakage in many brain regions (middle and right, Fig. 6D; quantified in Fig. 6N). Restricting Cas9 expression to endothelial cells (Fig. 6E) or using an alternative, single guide RNA (gRNA3, fig. S11, A to C) produced identical leakiness phenotypes (Fig. 6N), confirming the critical role of HYAL2 in maintaining the blood-brain barrier in adulthood. Similar to SLC7A1, loss of HYAL2 led to macromolecule leakage, including Evans blue-bound albumin (Fig. 6, D and E) and endogenous IgG (Fig. 6F). However, unlike SLC7A1, the leaking sites in *Hyal2* knockout expressed PLVAP (Fig. 6H) while retaining Claudin-5 expression (Fig. 6G), suggesting that HYAL2 loss induced, at least in part, fenestration of brain blood vessels.

Like mouse *Hyal2*, human *HYAL2* is expressed by brain endothelial cells at both developmental and adult stages, with the expression being higher in adults (Fig. 6, B and C). Unlike the age-specific effect of *Slc7a1* (Fig. 4 and fig. S9), knocking out *Hyal2* in neonatal mice caused similar blood-brain barrier leakage as adult knockout, using either gRNA1,2 or gRNA3 (Fig. 6, I and J and fig. S11, D and E; quantified in Fig. 6N). HYAL2 remained to be abundant at the vasculature luminal surface in aged mice (Fig. 6, A and B), sparking the question whether it also secures the blood-brain barrier in aging. The viral-genetic strategy (Fig. 6K) enabled *de novo* knockout in aged mice, which had unperturbed blood-brain barrier throughout development and adulthood, to specifically test the function of HYAL2 in aging, and also accelerated genetic causality test in an aging context as aged Cas9 mice can be pre-stocked even before the gene of interest is decided. Shown in Fig. 6L, control mice exhibited intact blood-brain barrier, highlighting its robustness in aging. Loss of HYAL2 caused leakage of both Evans blue (left, Fig. 6M; lightly blue-colored cortex) and Sulfo-NHS-LC-biotin (middle and right, Fig. 6M; quantified in Fig. 6N), albeit weaker leakiness than adult knockout. We note that aging alters chromatin accessibility (62), which may compromise Cas9 knockout efficiency.

Together, HYAL2 secures blood-brain barrier integrity from development through adulthood into aging.

## Structural and functional conservation of human and mouse HYAL2

**Fig. 7.**
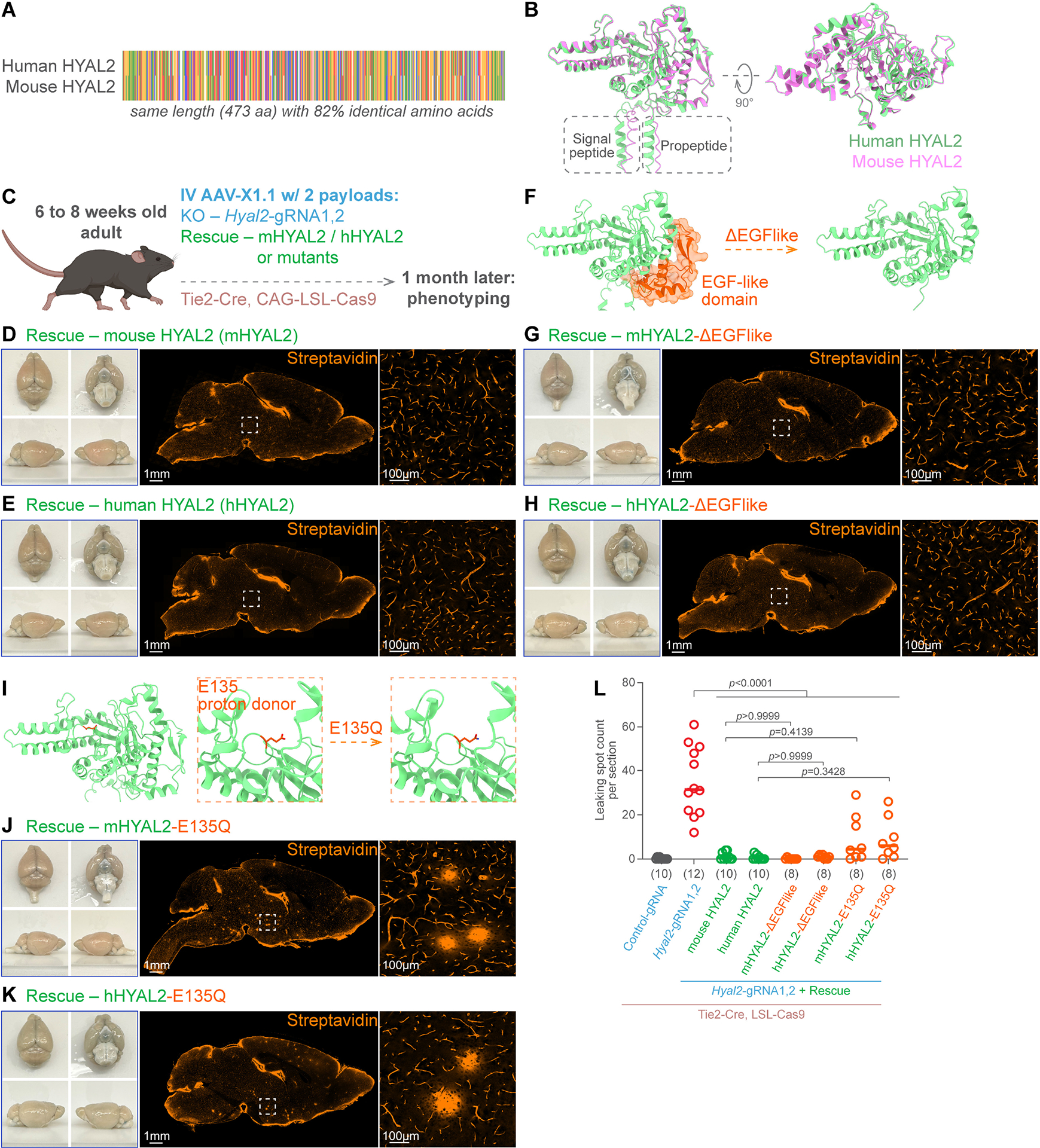
*In vivo* structure-function interrogation of human and mouse HYAL2. (**A**) Amino acid alignment of human and mouse HYAL2 proteins, using the Lesk color scheme. (**B**) Structure alignment of human and mouse HYAL2 proteins, based on AlphaFold (63, 64) predictions. (**C**) Schematic of the viral-genetic strategy for *in vivo* functional tests of mouse and human HYAL2, as well as their variants, in a rescue context. AAV-X1.1 that encodes two payloads—1) two *Hyal2*-targeting guide RNAs (*Hyal2*-gRNA1,2) for knockout and 2) the coding sequence of a HYAL2 variant for expression—is intravenously injected into 6 to 8 weeks old mice that express endothelial cell specific Cas9. One month after AAV injection, Evans blue is injected intraperitoneally and examined next day after cardiac perfusion of Sulfo-NHS-LC-biotin. (**D and E**) Genetic rescue of *Hyal2* knockout by mouse (D) or human (E) HYAL2. Left, whole brain photos showing the Evans blue stain. Middle, streptavidin staining of the brain sagittal section for Sulfo-NHS-LC-biotin detection. Right, zoom-in of the boxed region. (**F**) Predicted structures of wild-type (left) and EGF-like domain deleted (right) human HYAL2 proteins. (**G and H**) Genetic rescue of *Hyal2* knockout by EGF-like domain deleted mouse (G) and human (H) HYAL2 (ΔEGFlike). (**I**) Point mutation of the proton donor glutamic acid (E135) to glutamine (E135Q). (**J and K**) Genetic rescue of *Hyal2* knockout by proton donor mutated mouse (J) and human (K) HYAL2 (E135Q). (**L**) Quantification of Sulfo-NHS-LC-biotin leaking spots. The total number of mice used for each experimental condition, from left to right, was: 5, 6, 5, 5, 4, 4, 4, and 4. From each mouse, two sagittal brain sections were stained with streptavidin, imaged, and quantified. Numbers in parentheses indicate the total number of brain sections quantified for each condition. ANOVA with Tukey’s multiple comparison correction was used to calculate the *p* values.

Human and mouse HYAL2 proteins are highly conserved, with 82% of their amino acids being identical (Fig. 7A and fig. S12A). AlphaFold (63, 64) predictions also showed almost completely overlapping structures (Fig. 7B and fig. S12, B and C), suggesting prospective conservation of their *in vivo* functions. The viral-genetic strategy (Fig. 7C) allowed us to include a second payload, in the guide RNA encoded AAV-X1.1, for expressing human HYAL2, mouse HYAL2, or their variants and thus test their *in vivo* functionality in a genetic rescue context. Shown in Fig. 7, D and E, both mouse and human HYAL2 fully rescued the blood-brain barrier breakdown caused by *Hyal2* knockout, exhibiting no leakage of Evans blue or Sulfo-NHS-LC-biotin (compared with Fig. 6E; quantified in Fig. 7L), testifying the functional conservation of these HYAL2 orthologs.

Structural prediction revealed two prominent and conserved domains in human and mouse HYAL2: a catalytic cavity for hyaluronidase activity (65–67) and an EGF-like domain of unknown function, located on opposite sides of the protein (fig. S12, B and C). Given the common role of EGF-like domains in mediating cell-cell adhesion and signaling transduction, we tested whether the EGF-like domain is necessary for HYAL’s function at the blood-brain barrier. AlphaFold predicted that the EGF-like domain is folded independently, and its deletion does not disrupt the overall folding of the remaining part (right, Fig. 7F). Therefore, we deleted the entire EGF-like domains from mouse and human HYAL2 (ΔEGFlike). Complete rescue was observed by either mouse or human HYAL2-ΔEGFlike (Fig. 7, G and H), with no difference from full-length HYAL2 (Fig. 7L), indicating that the EGF-like domain is dispensable here.

HYAL2 belongs to the hyaluronidase family, which degrades hyaluronan polymers and thus remodels the extracellular matrix. *Hyal2* knockout in brain endothelial cells resulted in the accumulation of hyaluronan lining the blood vessels (fig. S11, F and G). Hyaluronidases share a conserved catalytic mechanism for cleaving hyaluronan, particularly the glutamic acid residue required as a proton donor to protonate the cleaved intermediate. Mutating this glutamic acid to glutamine, a similar amino acid without proton donor functionality due to the carboxyl-to-amide change, abolishes the enzymatic activity of hyaluronidases (65–67). To examine whether the enzymatic activity of HYAL2 is needed for securing blood-brain barrier integrity, we mutated the proton donor glutamic acid (E135) to glutamine (E135Q; Fig. 7I and fig. S12A). Mouse and human HYAL2-E135Q behaved similarly and failed to fully rescue the barrier breakdown caused by *Hyal2* knockout as seen by obvious yet weaker Sulfo-NHS-LC-biotin leakage (middle and right, Fig. 7, J and K; quantified in Fig. 7L) as well as minor Evan blue stain (left, Fig. 7, J and K). Together, these findings indicate that the enzymatic activity of HYAL2, but not its EGF-like domain, is necessary for the integrity of blood-brain barrier.

## Discussion

The identification of SLC7A1 and HYAL2 as blood-brain barrier regulators (Fig. 4 to 7) highlights that temporally-resolved proteomics not only uncovers the luminal surface remodeling and molecular dynamics of mouse brain vasculature across the lifespan (Fig. 2 and 3) but also enables *in vivo* functional discoveries, demonstrating the power of the method and its broad applicability to diverse organisms and biological questions (Fig. 1 and fig. S2).

## From vasculature luminal surface proteome to *in vivo* functional causality

Emerging proximity labeling technologies provide powerful tools for characterizing the cell-surface proteome in cultured cells and intact tissues (13–16, 68–70) and thus create opportunities to understand cell-cell communication in organismal physiology at a proteome scale. As a unique type of cell surface, the luminal surface of blood vessels serves as the interface of circulating blood and surrounding tissues and thus controls body-wide physiology (6). Exceptional heterogeneity and organ-specific properties of the vasculature system (4) are still challenging for *in vitro* models and demand *in vivo* methodologies. Labeling and capturing the luminal surface proteome *in vivo* (Fig. 1) paves the way to study a myriad of biological questions including: 1) the commonality and heterogeneity of vasculature luminal surface in distinct organs, as well as its functional orchestration with organotypic physiology; 2) vascular adaptation to aging, diet, exercise, and environmental factors; and 3) pathological remodeling of blood vessels in life-threatening diseases such as metastatic cancer and sepsis—prospectively informing novel molecular targets for drug development and organ-specific drug delivery. This method does not require genetic access and can be used on any organism with a circulatory system (for example, treeshrew; fig. S2), broadly expanding its utility from laboratory biomedical research to general biology, evolution, and ecology. For instance, proteomic profiling of animal species living at different altitudes may address how the vasculature system evolves to adapt to various oxygen concentrations.

Transcriptomic approaches (37, 58, 61, 71–77) have been widely applied to characterize gene expression profiles of endothelial cells from brain and other organs, as well as the cell types that interact with them, thereby revealing critical genes for blood–brain barrier formation, maintenance, and function. Complementing these transcriptomic studies, luminal surface proteomics directly measures cell surface proteins, whose trafficking and turnover are heavily regulated post-translationally and therefore often not well reflected by RNA amounts (14, 78–83). Moreover, the luminal surface comprises not only the membrane of endothelial cells but also the extracellular matrix and plasma-borne factors; consequently, its protein composition and dynamics reflects contributions from endothelial cells as well as from secreted proteins produced by other cell types.

Quantitative profiling of the mouse brain vasculature across development, adulthood, and aging (Fig. 2 and 3) uncovers the global reduction of angiogenesis factors and solute carrier transporters in the development-to-adult transition and themed transformations in aging: declined metabolic exchange, escalated pro-inflammatory signal, and impaired mechanical adaptation, as well as the key molecular underpinnings of these aging hallmarks (84). Following proteomics, *in vivo* genetic perturbation is needed to test functional causality of individual proteins. Our viral-genetic strategy enables age-specific and cell-type-specific knockout of any gene across the lifespan (Fig. 4 and 6), accelerating *in vivo* genetic interrogation compared with traditional genetic approaches particularly in aged mice. Moreover, it enables *in vivo* structure-function studies (Fig. 7), which are normally performed *in vitro* instead of *in vivo* due to technical difficulty and cost. With its conserved tropism in rodents (55), AAV-X1.1 has the potential to migrate such a versatile strategy and achieve *in vivo* functional causality tests in genetically non-tractable animals.

## Molecular mechanisms of blood-brain barrier integrity

Decades of research have identified several pivotal pathways underlying the formation and maintenance of the blood-brain barrier (19–21): Wnt-Frizzled-β-catenin axis (85–94), hedgehog signaling (95), tight junction assembly (96), transcytosis suppression (97, 98), and glycocalyx regulation (99). In this study, proteome-informed *in vivo* genetic screen identified two regulators of the blood-brain barrier: the amino acid transporter SLC7A1 and the hyaluronidase HYAL2 (Fig. 4 and 6). Two distinct permeability tracers, Sulfo-NHS-LC-biotin and Evans blue that binds to albumin, found that knockout of either gene led to leakage of both small chemical and protein. Nevertheless, their underlying mechanisms appear distinct. First, loss of HYAL2, but not SLC7A1, caused fenestration of brain blood vessels as shown by PLVAP staining, indicating distinct downstream effects. Second, HYAL2 is needed for the blood-brain barrier throughout the lifespan, whereas SLC7A1 is essential only in neonates, suggesting substantial differences between the neonatal and adult blood-brain barrier despite that the barrier is already established and functional in neonates.

SLC7A1 is a cationic amino acid transporter known to be amply expressed by brain endothelial cells (19, 58). Due to its molecular family, its primary function at the blood-brain barrier has long been presumed to involve the transport of amino acids to meet metabolic demands. It seems paradoxical that loss of such a transporter, which mediates permeation, increases permeability of the blood-brain barrier. Our further mechanistic analysis found that the nitric oxide synthase NOS3, which mirrors the expression pattern of SLC7A1 and is downstream of SLC7A1 in the nitric oxide synthesis pathway, is also essential for the blood-brain barrier during the neonatal stage but not in adulthood (Fig. 5), indicating that neonatal blood-brain barrier requires nitric oxide production, mediated by SLC7A1-driven arginine uptake and NOS3-dependent synthesis.

HYAL2 presents a paradox similar to SLC7A1. Hyaluronidases hydrolyze and thus lower the viscosity of hyaluronan, thereby increasing tissue permeability. Due to this effect, recombinant hyaluronidases are FDA-approved drugs to facilitate the dispersion and delivery of other drugs and currently marketed as Amphadase, Hydase, Hylenex, and Vitrase. In contrast, loss of hyaluronidase HYAL2 increases the permeability of blood-brain barrier, indicating that HYAL2 normally “seals” the barrier, opposite to its commonly recognized effect. We further found that loss of HYAL2 led to hyaluronan accumulation on the vascular luminal surface and that HYAL2’s hyaluronidase enzymatic activity is essential for the barrier integrity (Fig. 7), suggesting that blood-brain barrier requires HYAL2-mediated hyaluronan turnover.

Together, these findings highlight the signaling complexity underlying blood-brain barrier maintenance and functionally link the metabolism of nitric oxide and hyaluronan to blood-brain barrier integrity.

## Supplementary Material

aea2100_Suppl Excel_seq1_v2

aea2100_Suppl Excel_seq2_v2

aea2100_Suppl Excel_seq3_v2

aea2100_Suppl Excel_seq4_v2

aea2100_Suppl Excel_seq5_v1

aea2100_Suppl Excel_seq6_v1

aea2100_SupplementalMaterial_v4

Materials and Methods

Figs. S1 to S12

Tables S1 to S6

## Data Availability

The original mass spectra and the protein sequence database used for searches have been deposited in the public proteomics repository MassIVE and are accessible at ftp://MSV000097020@massive.ucsd.edu when providing the dataset password: WGA. If requested, also provide the username: MSV000097020. These datasets will be made public upon acceptance of the manuscript. Processed proteomic data is provided in table S1–S6. All reagents and materials generated in this study are available from the corresponding author upon request.
