# Supplementary material for: Luminal surface proteome of the brain vasculature uncovers blood-brain barrier regulators
Source: Science. Author manuscript; Available in PMC 2026 Jul 15. (PMC13372101; doi:10.1126/science.aea2100)
Supplement: aea2100_SupplementalMaterial_v4 [file NIHMS2191341-supplement-aea2100_SupplementalMaterial_v4.pdf]

Science
AAAS

Supplementary Materials for

Luminal surface proteome of the brain vasculature uncovers blood-brain barrier regulators

Zijian Zhu, Zuzhi Jiang, Yupu Wang, Khanh Nguyen, Yuxiang Zhang, Cameron Genxuan Lian, D. R. Mani, Jun Zheng, Lang Ding, Shihong Max Gao, Alps Xia, Anne Kuszpit, Sarah Lindo, Crystall Lopez, Catherine Lindsey, Brooke Groff, Xinhong Chen, Jiahui Wu, Weiliang Xia, Wei Li, Xiaorong Liu, Viviana Gradinaru, Steven A. Carr, Namrata D. Udeshi, Jiefu Li

Corresponding author: lij6@janelia.hhmi.org

The PDF file includes:

Materials and Methods
Figs. S1 to S12
Tables S1 to S6

Materials and Methods

Mouse

All mouse procedures followed protocols approved by the Institutional Animal Care and Use Committee of the Janelia Research Campus of the Howard Hughes Medical Institute. The following mouse strains were obtained from the Jackson Laboratory and maintained on a C57BL/6J background: C57BL/6J (Strain #000664), Tie2-Cre (Strain #008863) (*57*), CAG-Cas9 (Strain #026179) (*56*), and CAG-LSL-Cas9 (Strain #026175) (*56*). Male C57BL/6J mice were used for biotinylation, biochemical, and proteomic experiments. Mice of both sexes were used for *in vivo* genetic perturbation experiments.

Northern treeshrew

All treeshrew procedures followed the protocol approved by the Institutional Animal Care and Use Committee of the University of Virginia. Treeshrews were bred at the University of Virginia. Both sexes were used.

Study design and statistics

Sample sizes were not determined using statistical methods, but followed prior studies and convention in the field. Individual mice were randomly assigned to experimental conditions without prior selection. Data collection was not performed blind to experimental conditions. All samples and data were included in analysis without exclusion. Biological replicates, statistical methods, and results are reported in corresponding figure legends.

Biotinylation of vasculature luminal surface proteins in mice

For young adult (12-week-old) and aged (80-week-old) mice, terminal anesthesia was induced using isoflurane, followed by transcardial perfusion via the left ventricle using a 21-gauge needle. The right atrium was incised to allow outflow. Perfusion was carried out at room temperature at a flow rate of 10 mL/min, with the following solutions administered sequentially: 30 mL of phosphate-buffered saline, pH 7.4 (PBS; Gibco, 10010049), 100 mL of 250 nM horseradish peroxidase conjugated wheat germ agglutinin (WGA-HRP; Vector Laboratories, PL-1026-2) diluted in PBS, 100 mL of 100 μM biotin-xx-phenol (BxxP; APExBIO, A8012) and 1 mM hydrogen peroxide (H_2O_2; Thermo Scientific, H325-500) diluted in PBS, and 50 mL of the quencher solution containing 10 mM sodium ascorbate (Sigma-Aldrich, A4034), 5 mM Trolox (Sigma-Aldrich, 238813), and 10 mM sodium azide (Sigma-Aldrich, S2002) in PBS.

Step	Solution	Volume
1	PBS, pH 7.4 (all reagents below were diluted in PBS, pH 7.4)	30 mL
2	250 nM WGA-HRP	100 mL
3	100 μM BxxP + 1 mM H_2O_2	100 mL
4	Quencher (10 mM sodium ascorbate, 5 mM Trolox, 10 mM sodium azide)	50 mL

For 14-day-old neonatal mice, terminal anesthesia was induced using isoflurane, followed by transcardial perfusion via the left ventricle using a 23-gauge needle. The right atrium was incised to allow outflow. Perfusion was carried out at room temperature at a flow rate of 6.7 mL/minute with the following solutions administered sequentially: 15 mL of PBS (Gibco, 10010049), 40 mL of 250 nM WGA-HRP (Vector Laboratories, PL-1026-2), 40 mL of 100 μM

BxxP (APExBIO, A8012) and 1 mM H_2O_2 (Thermo Scientific, H325-500), and 20 mL of the quencher solution containing 10 mM sodium ascorbate (Sigma-Aldrich, A4034), 5 mM Trolox (Sigma-Aldrich, 238813), and 10 mM sodium azide (Sigma-Aldrich, S2002). For '–WGA' controls, PBS was perfused instead of WGA-HRP.

Step	Solution	Volume
1	PBS, pH 7.4 (all reagents below were diluted in PBS, pH 7.4)	15 mL
2	250 nM WGA-HRP	40 mL
3	100 μM BxxP + 1 mM H_2O_2	40 mL
4	Quencher (10 mM sodium ascorbate, 5 mM Trolox, 10 mM sodium azide)	20 mL

For biochemical experiments and proteomic sample collection, tissues were immediately dissected into Protein LoBind tubes (Eppendorf, 022431081) and snap-frozen in liquid nitrogen before storage at –80°C. For histology, mice were further perfused with 4% paraformaldehyde (PFA; EMS, 15714-S) diluted in PBS before tissue collection.

We note that peroxidase-mediated cell-surface biotinylation (*13–16*), including the method reported in this study, relies on hydrogen peroxide (H_2O_2). Although the concentration used is low (0.003%, 1 mM) and the exposure is brief before immediate quenching, H_2O_2 may still perturb the redox environment and potentially alter protein modifications.

Biotinylation of vasculature luminal surface proteins in treeshrews

For treeshrews, terminal anesthesia was induced using isoflurane, followed by transcardial perfusion via the left ventricle using a 19-gauge needle. The right atrium was incised to allow outflow. Perfusion was carried out at room temperature at a flow rate of 50 mL/minute with the following reagents: 50 mL of PBS (Gibco, 10010049), 300 mL of 82 nM WGA-HRP (Vector Laboratories, PL-1026-2), 300 mL of 100 μM BxxP (APExBIO, A8012) and 1 mM H_2O_2 (Thermo Scientific, H325-500), 200 mL of the quencher solution containing 10 mM sodium ascorbate (Sigma-Aldrich, A4034), 5 mM Trolox (Sigma-Aldrich, 238813), and 10 mM sodium azide (Sigma-Aldrich, S2002), and 300 mL of 4% PFA (EMS, 15714-S). For the '–WGA' control, PBS was perfused instead of WGA-HRP.

Step	Solution	Volume
1	PBS, pH 7.4 (all reagents below were diluted in PBS, pH 7.4)	50 mL
2	82 nM WGA-HRP	300 mL
3	100 μM BxxP + 1 mM H_2O_2	300 mL
4	Quencher (10 mM sodium ascorbate, 5 mM Trolox, 10 mM sodium azide)	200 mL

Biotinylation by Sulfo-NHS-LC-biotin perfusion in mice

To test whether Sulfo-NHS-LC-biotin can be used to biotinylate vasculature luminal surface proteins *in vivo* via perfusion (fig. S5), young adult (12-week-old) mice were terminally anesthetized using isoflurane, followed by transcardial perfusion via the left ventricle using a 21-gauge needle. The right atrium was incised to allow outflow. Perfusion was carried out at room temperature at a flow rate of 10 mL/min, with the following solutions administered sequentially: 20 mL of PBS (Gibco, 10010049), 30 mL of 0.5 mg/mL Sulfo-NHS-LC-biotin (Thermo Scientific, 21335), 30 mL of Tris-buffered saline (Thermo Scientific, 28376) that quenches Sulfo-NHS-LC-biotin, and 40 mL of 4% PFA (EMS, 15714-S).

Histology

Tissues were post-perfusion fixed in 4% PFA (EMS, 15714-S) for two overnights on a shaker at 4°C, rinsed with PBS, and then sectioned on a vibratome (Leica, VT1200S) for 50 µm sections. Tissue sections were incubated in the blocking solution containing 5% normal donkey serum (Jackson ImmunoResearch, 017-000-121) and 0.1% Triton X-100 (Sigma-Aldrich, T9284) in PBS (Gibco, 10010049) for two hours or longer on an orbital shaker at room temperature. For anti-PECAM1 staining, the goat anti-PECAM1 antibody (R&D Systems, AF3628) was 500-fold diluted in the blocking solution and incubated with tissue sections for two overnights on an orbital shaker at 4°C, followed by secondary antibody staining. For anti-PLVAP staining, the rat anti-PLVAP antibody (BD, 553849) was 200-fold diluted in the blocking solution and incubated with tissue sections for two overnights on an orbital shaker at 4°C, followed by secondary antibody staining. For anti-Claudin-5 staining, the mouse anti-Claudin-5 antibody (Invitrogen, 35-2500) was first conjugated to the Alexa Fluor 647 dye (Invitrogen, A88068) and then 100-fold diluted in the blocking solution and incubated with tissue sections for two overnights on an orbital shaker at 4°C. For hyaluronan detection, biotinylated hyaluronan binding protein (Sigma-Aldrich, 385911) was diluted in the blocking solution to 2.5 µg/mL and incubated with tissue sections for two overnights on an orbital shaker at 4°C, followed by streptavidin staining. For secondary antibody staining, after three rounds of washes in 0.1% Triton X-100 (Sigma-Aldrich, T9284) at room temperature, tissue sections were incubated in fluorophore-conjugated, species-specific anti-IgG antibodies (Jackson ImmunoResearch) 250-fold diluted in the blocking solution for two overnights on an orbital shaker at 4°C. For streptavidin staining, fluorophore-conjugated streptavidin proteins (Invitrogen) were 500-fold diluted in the blocking solution solely (for streptavidin-only staining) or together with the secondary antibodies and incubated with tissue sections for two overnights on an orbital shaker at 4°C. For mounting, after three rounds of washes in 0.1% Triton X-100 (Sigma-Aldrich, T9284) at room temperature and a quick rinse in PBS, tissue sections were mounted in a DAPI-containing mounting solution (Vector Laboratories, H-1800) and stored at 4°C.

Tissue homogenization and lysis

Frozen tissue samples (~200 mg each) were transferred into pre-cooled tubes containing 1 mL of 1x RIPA lysis buffer (Sigma-Aldrich, 20188), 10 g/L sodium dodecyl sulfate (SDS; Sigma-Aldrich, L6026), and 1x protease inhibitor cocktail (Thermo Scientific, 78438). A refrigerated homogenizer (Benchmark, D2400-R) and 1.5 mm diameter zirconium beads (Benchmark, D1032-15) were used to homogenize and lyse tissue samples at 4,200 rpm and 4°C, using a 30-cycle program that consists of 30-second cooling intervals between each 30-second operation. Following the removal of zirconium beads, 4 mL of 1x RIPA lysis buffer (Sigma-Aldrich, 20188) supplemented with 1x protease inhibitor cocktail (Thermo Scientific, 78438) was added to each 1 mL of sample to reduce the SDS concentration to 2 g/L. Samples were then sonicated on ice bath at 4°C, using a probe sonicate (Benchmark, DP0150) and a 15-minute, 45-watt program that consists of 15-second cooling intervals between each 30-second operation. Tissue lysates were then rotated at 4°C for two hours before ultracentrifugation at 100,000 g and 4°C for 40 minutes. Clear supernatant from each sample was carefully collected, avoiding the cloudy lipid layer at the top and the precipitate at the bottom, which can severely interfere with streptavidin bead enrichment.

Streptavidin bead enrichment of biotinylated proteins

Total protein concentrations of samples were measured using the Qubit Protein Broad Range Assay (Invitrogen, A50669) and normalized to 3 mg/mL by diluting with 1x RIPA lysis buffer (Sigma-Aldrich, 20188) supplemented with 1x protease inhibitor cocktail (Thermo Scientific, 78438). 75 μL of streptavidin bead slurry (Pierce, 88817) was used to enrich biotinylated proteins from 1 mg of total proteins. For each proteomic sample, 300 μL of bead slurry was used to enrich biotinylated proteins from 4 mg of total proteins. Streptavidin beads were first washed twice in 1 mL of 1x RIPA lysis buffer (Sigma-Aldrich, 20188) and then incubated with tissue lysates on a rotator for one overnight at 4°C. Post-enrichment beads were washed sequentially by two rounds of 1x RIPA lysis buffer (Sigma-Aldrich, 20188), one round of 1 M potassium chloride (Sigma-Aldrich, P9333), one round of 100 mM sodium carbonate (Sigma-Aldrich, S7795), one round of 2 M urea (Sigma-Aldrich, 51456) in 10 mM Tris-HCl, pH 8.0 (Invitrogen, 15568025), and two more rounds of 1x RIPA lysis buffer (Sigma-Aldrich, 20188). All these wash solutions were supplemented with 1x protease inhibitor cocktail (Thermo Scientific, 78438). Beads were then washed three rounds by PBS (Gibco, 10010049) without protease inhibitors and transferred to new tubes. 10 μL of bead slurry was collected from each sample for quality control (fig. S4B; fig. S6, C to E).

Streptavidin and western blot

4% to 12% Bis-Tris polyacrylamide gels (Invitrogen, NW04125BOX) and polyvinylidene fluoride transfer stacks (Invitrogen, IB24001) were used for protein electrophoresis and transfer, following the manufacturer's protocols. Post-transfer membranes were blocked in the SuperBlock buffer (Thermo Scientific, 37536) for 30 minutes or longer on an orbital shaker at room temperature. For streptavidin blot, membranes were incubated with horse-radish peroxidase (HRP) conjugated streptavidin (Thermo Scientific, N100) 2,500-fold diluted in the SuperBlock solution (Thermo Scientific, 37536) for one overnight on an orbital shaker at 4°C. For western blot, membranes were incubated with primary antibodies diluted in the SuperBlock solution (Thermo Scientific, 37536) for one overnight on an orbital shaker at 4°C, washed four rounds in Tris-buffered saline containing 0.05% Tween-20 (Pierce, 28360), and then incubated with HRP-conjugated secondary antibodies (Jackson ImmunoResearch) for one hour or longer on an orbital shaker at room temperature. After four rounds of washes in Tris-buffered saline containing 0.05% Tween-20 (Pierce, 28360), chemiluminescence was developed using the Clarity Western ECL substrate (Bio-Rad, 1705060) and imaged using an Amersham ImageQuant 800 imaging system (Cytiva).

Primary antibodies used for western blot in this study included goat anti-PECAM1 (1:500; R&D Systems, AF3628), rabbit anti-ATP1A1 (1:20,000; Abcam, ab76020), mouse anti-GAPDH (1:20,000; Invitrogen, MA5-15738), mouse anti-β-actin (1:20,000; Invitrogen, MA5-15739), and rabbit anti-lamin A/C (1:5,000; Invitrogen, MA5-35284). HRP-conjugated secondary antibodies (Jackson ImmunoResearch) were used at 1:10,000.

On-bead trypsin digestion of biotinylated proteins

For both brain and kidney samples, biotinylated proteins bound to streptavidin magnetic beads were washed four times with 200 μL of 50 mM Tris-HCl (pH 7.5) buffer. The final wash was then removed and 80 μL of the digestion buffer (2 M urea, 50 mM Tris-HCl, 1 mM dithiothreitol, and 0.4 μg of trypsin) was added for incubation at room temperature while shaking at 1,000 rpm. Incubation was conducted for 1 hour and the supernatant was collected and transferred into a separate tube. This process was then repeated for another round with 30-minute incubation. After

the second supernatant was collected, 60 µL of 2 M urea and 50 mM Tris-HCl buffer was added to the tube for two rounds of washes, which were collected and pooled with the digestion supernatant. The pooled eluate was spun down at 5,000 g for 30 seconds. Next, the supernatant was collected and reduced with 4 mM dithiothreitol for 30 minutes, followed by alkylation with 10 mM iodoacetamide for 45 minutes in the dark at room temperature while shaking at 1,000 rpm. Finally, each sample was further digested overnight with 0.5 µg of trypsin on a shaker at room temperature and 1,000 rpm.

The following morning, neat formic acid (FA) was used to acidify digested peptide samples to the final concentration of 1% FA (pH < 3). Samples were then desalted using in-house packed C18 StageTips. Briefly, C18 StageTips were conditioned sequentially with 100 µL of 100% methanol (MeOH), 100 µL of 50% acetonitrile (MeCN) with 0.1% FA, and two rounds of 100 µL of 0.1% FA. Next, acidified peptides were loaded onto the C18 StageTips and washed twice with 100 µL of 0.1% FA. Desalted peptides were then eluted from the C18 resin using 50 µL of 50% MeCN/0.1% FA, snap-frozen, and vacuum-centrifuged until completely dry.

TMT labeling and StageTip peptide fractionation

Peptides were resuspended in 80 µL of 50 mM HEPES. The brain samples were labelled with 20 µL of 25 µg/µL TMTpro reagents in MeCN. The kidney samples were labelled with 25 µL of 20 µg/µL TMT 6-plex reagents in MeCN. Samples were then incubated for 1 hour at room temperature while shaking at 1,000 rpm. TMT labeling reactions were quenched by incubating with 4 µL of 5% hydroxylamine for 15 minutes at room temperature with 1,000 rpm shaking. Next, TMT-labelled peptides from all samples were pooled into one tube, snap-frozen, and vacuum-centrifuged to dry. The samples were then reconstituted in 200 µL of 1% FA, desalted on a C18 StageTip using the previously described protocol, and dried to completion.

The labelled peptides underwent basic reverse phase (bRP) fractionation on an in-house packed SDB-RPS StageTip. Specifically, the SDB-RPS StageTip was conditioned sequentially with 100 µL of 100% MeOH, 100 µL of 50% MeCN/0.1% FA, and two rounds of 100 µL of 0.1% FA. Peptides were resuspended in 200 µL of 0.1% FA, loaded on the conditioned StageTip, and then eluted from the StageTip in eight fractions using 20 mM ammonium formate buffers with increasing concentrations of MeCN (5%, 7.5%, 10%, 12.5%, 15%, 20%, 25%, and 45%). The eight fractions were then vacuum-centrifuged until completely dry.

Liquid chromatography and mass spectrometry

Peptide samples of the brain were analyzed on an online liquid chromatography tandem mass spectrometry (LC-MS/MS) system, including a Vanquish Neo UPHLC (Thermo Fisher) coupled to an Orbitrap Exploris 480 (Thermo Fisher). Previously collected eight fractions were reconstituted in 9 µL of 3% MeCN/0.1% FA, and 4 µL of each fraction was injected onto an in-house packed microcapillary column (Picofrit with 10 µm tip opening and 75 µm diameter; New Objective, PF360-75-10-N-5) with 30 cm of C18 silica material (1.5 µm ReproSil-Pur C18-AQ medium; Dr. Maisch GmbH, r119.aq), heated to 50°C using column heater sleeves (PhoenixST). Peptides were eluted into the Orbitrap Exploris 480 at a flow rate of 200 nL/min. Each fraction was run on a 110-minute method, including a linear 84-minute gradient from 94.6% solvent A (0.1% FA) to 27% solvent B (99.9% MeCN, 0.1% FA), followed by a linear 9-minute gradient from 27% solvent B to 54% solvent B.

Mass spectrometry was conducted in a data-dependent acquisition mode. MS1 spectra were measured with 60,000 resolution, 300% normalized AGC target, and m/z range from 350 to 1800.

MS2 spectra were acquired for the top 20 most abundant ions per cycle at 45,000 resolution, 30% AGC target, 0.7 m/z isolation window, and 34 normalized collision energy. The dynamic exclusion time was set to 20 seconds, and the peptide match and isotope exclusion functions were enabled.

Kidney samples were analyzed using the same LC-MS/MS system and acquisition parameters as described above, with a few key modifications: peptides were eluted into the Orbitrap Exploris 480 at a pressure of 600 bar; MS2 spectra were acquired at 15,000 resolution with other parameters remaining the same.

Mass spectrometry data processing

Mass spectrometry data of the brain samples were processed using Spectrum Mill Rev BI.07.11.216 (proteomics.broadinstitute.org). Raw file extraction retained spectra within a precursor mass range of 600-6000 Da with a minimum MS1 signal-to-noise ratio of 25. Additionally, MS1 spectra within a retention time range of +/- 45 s or within a precursor m/z tolerance of +/- 1.4 m/z were merged. MS/MS searching was performed against the UniProt mouse database, released on April 7, 2021. Digestion parameters were set to “trypsin allow P” with an allowance of 4 missed cleavages. The MS/MS search included fixed modifications, carbamidomethylation on cysteine and TMTPro on the N-terminus and internal lysine, and variable modifications, acetylation of the protein N-terminus, oxidation of methionine, N-terminal Q-pyroglutamate formation, and N-terminal deamidation. The matching tolerances were set with a minimum matched peak intensity of 30%, precursor and product mass tolerance of +/- 20 ppm, and a maximum ambiguous precursor charge of 3. Peptide spectrum matches were validated with a maximum false discovery rate threshold of 1.2% for precursor charges ranging from +2 to +6. A target protein score of 0 was applied during protein polishing auto-validation to further filter peptide spectrum matches. TMT reporter ion intensities were corrected for isotopic impurities using the afRICA correction method in the Spectrum Mill protein/peptide summary module, which utilizes determinant calculations according to Cramer's Rule. Protein quantification and statistical analysis were performed using the Proteomics Toolset for Integrative Data Analysis (Protigy, v1.0.7; Broad Institute, https://github.com/broadinstitute/protigy). Each protein was associated with a $\log_2$-transformed ratio of every TMT condition to the median intensity of all channels. Protein data were then normalized to the median within two separate groups: +WGA (WGA-HRP perfused, biotinylated) and –WGA (WGA-HRP omitted, control).

Mass spectrometry data of the kidney samples were processed using the same parameters on Spectrum Mill as described above, with one key modification: the MS/MS search included carbamidomethylation on cysteine and TMT6 on the N-terminus and internal lysine as fixed modifications. Additionally, protein quantification and statistical analysis were performed using Protigy v1.1.8.

Linear model for the mass spectrometry data

For the brain samples, a linear model was fit to model protein abundance across ages—postnatal day 14, 12 weeks old, and 80 weeks old—for each protein. The model also incorporated, via an interaction term, the +WGA (WGA-HRP perfused, biotinylated) and –WGA (WGA-HRP omitted, control) categories within each age to control for non-specific capturing effects including endogenously biotinylated proteins, non-specific bead binders, and other contaminants:

Protein abundance ~ age * WGA category.

Appropriate contrasts were then calculated to assess differential protein expression between any two ages. The linear model was implemented using the limma R package (*100*). The

resulting nominal p values for all proteins were adjusted for multiple testing using the Benjamini-Hochberg false discovery rate correction (*101*).

Proteomic data analysis

To remove intracellular contaminants, we performed cutoff analyses of the brain data using two different methods and obtained largely overlapping results (fig. S6J; table S2 and S3). Method #1 followed the previously described ratiometric strategy (*13–15*, *24*, *25*). Proteins of each age were ranked in descending order by the biotinylated-to-control ('+WGA' to '–WGA') ratio. Proteins with cell-surface annotations were labelled as true-positives, specified by the UniProt term: *((cc_scl_term:SL-0112) OR (cc_scl_term:SL-0243) OR (keyword:KW-0732) OR (cc_scl_term:SL-9906) OR (cc_scl_term:SL-9907)) AND (reviewed:true)*, which includes SwissProt-reviewed extracellular (SL-0112), secreted (SL-0243), signal peptide-containing (KW-0732), type II transmembrane (SL-9906), and type III transmembrane (SL-9907) proteins. Proteins with either nuclear (SL-0191), mitochondrial (SL-0173), or cytoplasmic (SL-0091) annotations but without cell-surface annotations were classified as false-positives, defined by the UniProt term: *(((cc_scl_term:SL-0091) OR (cc_scl_term:SL-0173) OR (cc_scl_term:SL-0191)) AND (reviewed:true)) NOT (((cc_scl_term:SL-0112) OR (cc_scl_term:SL-0243) OR (keyword:KW-0732) OR (cc_scl_term:SL-9906) OR (cc_scl_term:SL-9907)) AND (reviewed:true))*. For the top 1,000 proteins of each age, the accumulative true positive rates and false positive rates were calculated along the ranked list and a cutoff position was set where the value of 'true positive rate – false positive rate' was maximal. Proteins ranked above this cutoff position were retained for each age. We note that only the top 1,000 instead of the total 4,528 protein were used for cutoff position determination for two reasons: 1) Because of on-bead trypsin digestion, peptides of non-specific bead binders were released and detected. These low-abundance contaminants constituted the majority of lower-ranked proteins and generated stochastic noise interfering with the determination of cutoffs; and 2) Specific cell types or compartments are typically expected to express only a few hundred distinct cell-surface proteins. The cutoff results obtained using Method #1 are reported in Fig. 2, D to F and table S2. Method #2 used a fixed retaining criterion for all ages: $\log_2$(+WGA/–WGA fold change) > 0.5 and $p < 0.05$, and obtained the cutoff results reported in fig. S6, N and O and table S3. While Method #2, which uses a fixed fold-change cutoff, is commonly applied in proteomic and transcriptomic data analysis, its threshold is an arbitrary value determined manually. In contrast, Method #1 determines its cutoff dynamically based on the data's own performance—specifically, at the point where the separation between signal (true positives) and noise (false positives) is maximized. This approach provides a more objective and data-driven threshold for cutoff. The kidney data was analyzed using Method #1 and reported in fig. S4 and table S6. Gene ontology analyses were performed via the Panther web portal (https://www.pantherdb.org/) (*102*, *103*). Top ten "cellular component" (location) and "biological process" (function) terms with the lowest false discovery rates were plotted.

CRISPR guide RNA design and constructs

Guide RNAs (gRNAs) were designed using the CHOPCHOP web portal (https://chopchop.cbu.uib.no/) (*104–106*):

Control-gRNA1: 5'- GCGGCGTATTTATCCACCAA (no predicted target in the mouse genome)
Control-gRNA2: 5'- TCGCAGATGGTCGCTGCCCT (no predicted target in the mouse genome)
Slc7a1-gRNA1: 5'- TGACGTGAGAACTCTCCGAT
Slc7a1-gRNA2: 5'- TCTACAGCTACGTGACGGTG

Slc7a1-gRNA3: 5'- GTGGTGTCCGGGTTCGTGAA
Hyal2-gRNA1: 5'- TGCCAGTTTCGAACCCATAC
Hyal2-gRNA2: 5'- CACGGGCCGCTGTCCCGATG
Hyal2-gRNA3: 5'- ACGCGTCCCACATACACCCG
Slc16a1-gRNA1: 5'- ACTGGCCCTCCAATCGCAGG
Slc16a1-gRNA2: 5'- AGCCGTCCAGTAATGATCGC
Slc5a6-gRNA1: 5'- GCAGTTCACCAACGGTATGG
Slc5a6-gRNA2: 5'- GCATCTGACCAGCGCCTATG
Slc3a2-gRNA1: 5'- GGTCCGGTATAAAGTCTGCG
Slc3a2-gRNA2: 5'- GGTCAACAGCAGGTCGCTGG
Tfrc-gRNA1: 5'- TGTTGCGGCGAAGTCCAGTG
Tfrc-gRNA2: 5'- CTGCTGGGTCTAAATCCATC
Prom1-gRNA1: 5'- CATTCGGCTGGACCACGTTG
Prom1-gRNA2: 5'- TGGAAGAATATGACTCGTAC
Mpzl1-gRNA1: 5'- CGGGGCCGCAATCAGTGCCA
Mpzl1-gRNA2: 5'- TCGGTGCTAGCCGCGATGCT
Scarf1-gRNA1: 5'- TGGCATGGGCGACGACACGT
Scarf1-gRNA2: 5'- ACTGCGACCCCGGTTGGCTG
Serpina1d-gRNA1: 5'- CCAGCTCCCTGTATAGTCTG
Serpina1d-gRNA2: 5'- GATATGGATCTGAGCATCGC
Nos3-gRNA1: 5'- GGATCCCAAGCAGCGTCTTG
Nos3-gRNA2: 5'- CGCCGCCAAGAGGATACCAG

To express gRNAs *in vivo* using adeno-associated viruses (AAVs), we built an AAV vector comprising two gRNA loci driven by U6 promoters and one StayGold fluorescent protein (*107*) driven by a EF1α short promoter between the AAV2 inverted terminal repeats. Before gRNA insertion, the loci contain octuple thymidines to stop the expression of empty gRNA scaffolds. SapI (NEB, R0569) and BbsI (NEB, R3539) were used to cut these two gRNA loci, respectively, for gRNA insertion by the NEBuilder HiFi DNA Assembly Master Mix (NEB, E2621). For rescue experiments, coding sequences of human and mouse *Hyal2* were cloned from human and mouse brain cDNA libraries (Zyagen, MD-201-BLC and MD-201-C57), respectively, using the Q5 DNA Polymerase (NEB, M0493). Mutations and deletions were introduced using the Q5 Site-Directed Mutagenesis Kit (NEB, E0554). The coding sequences of human *HYAL2*, mouse *Hyal2*, and their variants were cloned into the *Hyal2*-gRNA1,2 AAV vector to replace the StayGold coding sequence. NEB Stable Competent *E. coli* (NEB, C3040) cultured at 30°C was used to minimize vector recombination. All plasmids were whole-plasmid sequenced by Plasmidsaurus.

Adeno-associated virus

AAVs with the X1.1 capsid (*55*) or the PHP.eB capsid (*108*) were produced by the Viral Tools team of the Janelia Research Campus and used following protocols approved by the Institutional Animal Care and Use Committee. For newborn mice on post-natal day 0, 7.5e10 vg (viral genome) of AAV was injected intravenously via the temporal vein using 32-gauge needles and 25 uL Hamilton syringes (Hamilton, 7803-14 and 7636-01). For young adult (12-week-old) and aged (80-week-old) mice, 1e12 vg of AAV was injected intravenously into the retro-orbital sinus using 31-gauge, 300 uL insulin syringes (BD, 328440).

Permeability tests of the blood-brain barrier

Evans blue (Sigma-Aldrich, E2129) was dissolved in PBS (Gibco, 10010049) at the concentration of 10 mg/mL and sterilized by filtering through 0.22 µm pore size PVDF membranes (Millipore, SE1M179M6). For neonatal mice injected with AAVs on postnatal day 0, Evans blue was injected intraperitoneally at the dose of 60 µg per gram of body weight on postnatal day 13. On postnatal day 14, mice were terminally anesthetized using isoflurane and perfused at the speed of 6.7 mL/minute with the following reagents: 15 mL of PBS (Gibco, 10010049), 15 mL of 0.5 mg/mL Sulfo-NHS-LC-biotin (Thermo Scientific, 21335) dissolved in PBS (Gibco, 10010049), 15 mL of Tris-buffered saline (Thermo Scientific, 28376) that quenches Sulfo-NHS-LC-biotin, and 20 mL of 4% PFA (EMS, 15714-S). For young adult and aged mice, Evans blue was injected intraperitoneally at the dose of 180 µg per gram of body weight one month after AAV injection. Next day, mice were terminally anesthetized using isoflurane and perfused at the speed of 10 mL/minute with the following reagents: 20 mL of PBS (Gibco, 10010049), 30 mL of 0.5 mg/mL Sulfo-NHS-LC-biotin (Thermo Scientific, 21335), 30 mL of Tris-buffered saline (Thermo Scientific, 28376), and 40 mL of 4% PFA (EMS, 15714-S). Immediately after perfusion, brains were dissected and photographed using an iPhone (Apple). Staining of brain sagittal sections followed the procedures outlined in the Histology section above.

Confocal microscopy

A TissueFAXS SL upright confocal slide scanner (TissueGnostics) with a 60 µm spinning disk and a 20x/0.8 air objective was used to acquire images. The TissueFAXS SL Viewer software (TissueGnostics) was used to export images. From confocal images of brain sagittal sections, Sulfo-NHS-LC-biotin leaking spots were counted.

Quantitative RT-PCR

To validate CRISPR/Cas9-mediated gene knockout *in vivo*, we used AAV-PHP.eB (*108*) to deliver guide RNAs listed above to adult CAG-Cas9 mice, which express body-wide Cas9 (*56*). For *Nos3*, we used AAV-X1.1 (*55*) and endothelial cell specific Cas9 (Tie2-Cre (*57*), CAG-LSL-Cas9 (*56*)) instead due to its known endothelial cell specific expression. Each mouse received 1e12 vg (viral genome) of AAV administered via intravenous injection into the retro-orbital sinus using 31-gauge, 300 µL insulin syringes (BD, 328440). One month post-injection, brains were collected for total RNA extraction using the Direct-zol RNA Miniprep Kit (Zymo, R2053) following the manufacturer's protocol. Quantitative RT-PCR was performed using the Luna Universal One-Step RT-qPCR Kit (NEB, E3005E) on a QuantStudio 3 Real-Time PCR System (Thermo Scientific). Gene-specific primers without any predicted off-targets were designed by Primer-BLAST (https://www.ncbi.nlm.nih.gov/tools/primer-blast/):

Actb (for normalization):
5'- GATTACTGCTCTGGCTCCTAG and 5'- GACTCATCGTACTCCTGCTTG

Slc7a1 (primer pair 1):
5'- CTCAGCCTACCTCTACAGCTA and 5'- CTTGCCTATCAGCTCGTCAA

Slc7a1 (primer pair 2):
5'- TACAGCTACGTGACGGTGGG and 5'- AGAACTCTCCGATGGGCTTG

Hyal2 (primer pair 1):
5'- GATCGGGCTTGGTTGGTA and 5'- AGTGTGATGATGGGACCTAGT

Hyal2 (primer pair 2):
5'- GAATGGCGGCCTGTATGGGT and 5'- TCCACATCGGGACAGCGG

Slc16a1 (primer pair 1):
5'- GCCGTCCAGTAATGATCGC and 5'- GTTGAAAGCAAGCCCAAGACC
Slc16a1 (primer pair 2):
5'- AAAATGCCACCTGCGATTGGA and 5'- GACAACCACCAGCGATCATT
Slc5a6 (primer pair 1):
5'- GGGGCCACCATACCGTT and 5'- AGCACCCCAGGAACCAATAC
Slc5a6 (primer pair 2):
5'- TTCCTGGGGTGCTCCTACTT and 5'- TAGGTACTCATAGGCGCTGGT
Slc3a2 (primer pair 1):
5'- CCTCGCAGACTTTATACCGGAC and 5'- GGCCCTTCACTGTCATGTTG
Slc3a2 (primer pair 2):
5'- ACGGTGTGGATGGTTTCCAA and 5'- TCAACAGCAGGTCGCTGG
Tfrc (primer pair 1):
5'- TCTCAGTCATCAGGGTTGCC and 5'- CCCACACTGGACTTCGCC
Tfrc (primer pair 2):
5'- GCGAAGTCCAGTGTGGGAA and 5'- TGCTGGGTCTAAATCCATCTTTT
Prom1 (primer pair 1):
5'- CTGGGGCTGTGTGGAAAGAT and 5'- CTGGACCACGTTGAGGAAGA
Prom1 (primer pair 2):
5'- TGACTCGTACTGGTGGCTG and 5'- GCCTCTTCTAGTTGGGGTGG
Mpzl1 (primer pair 1):
5'- CCGTGGCACTGATTGCGG and 5'- ACCTGTCCTTGTGAGTAGTGG
Mpzl1 (primer pair 2):
5'- GTGTGGTCTAAGGCAAGCCA and 5'- TGTCAACAGCCCGAGCATC
Scarf1 (primer pair 1):
5'- CACCCACGTGTCGTCGC and 5'- CACACACTGGCACTTACGAC
Scarf1 (primer pair 2):
5'- TGAATGCCACATGCTCTCCA and 5'- CCTGTGCCCCAGCCAAC
Serpina1d (primer pair 1):
5'- GGCTGAGGATGTTCAGGAGAC and 5'- CCCTGTATAGTCTGAGGGCAAA
Serpina1d (primer pair 2):
5'- AAACAGGCGCAGAAGCGAT and 5'- GAGAGGTCAGCCCCATTGTT
Nos3 (primer pair 1):
5'- CTGTACCTCAAGACGCTGCT and 5'- CCTGGGAGCCACTCCTTTTG
Nos3 (primer pair 2):
5'- GGTCCTGTGCATGGATGAGT and 5'- GCCGCCAAGAGGATACCAG

Protein structure prediction and visualization

Protein structures were predicted by AlphaFold (https://alphafoldserver.com/) (*63*, *64*) and visualized in ChimeraX (*109*).

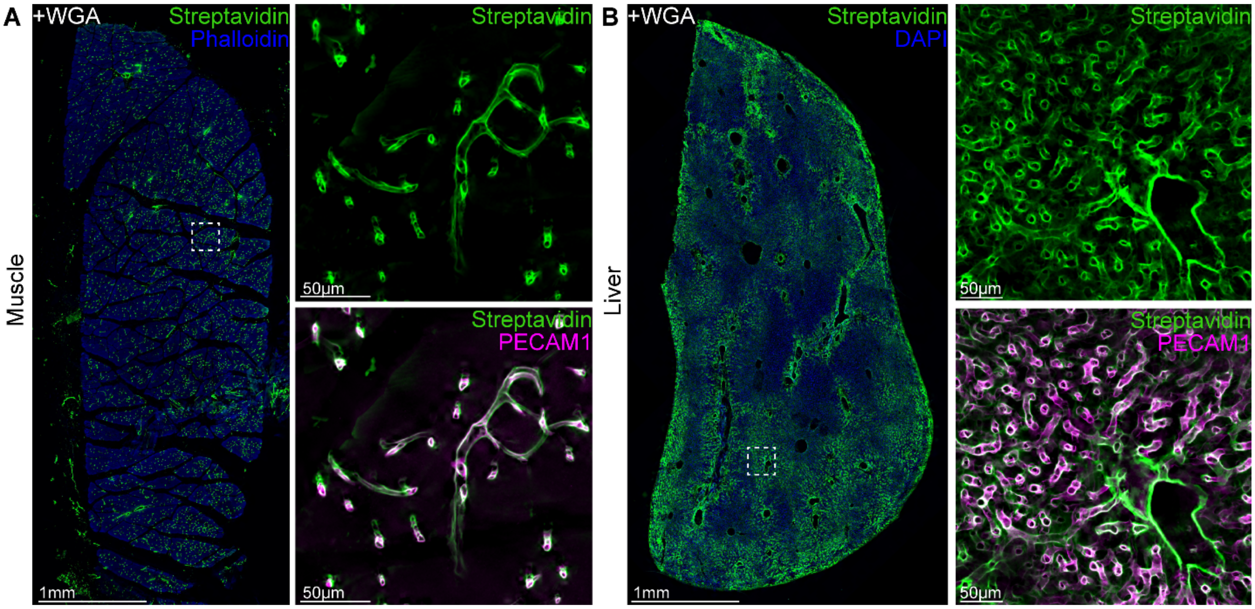

Fig. S1. Biotinylation of the vasculature luminal surface in muscle and liver.
Streptavidin and anti-PECAM1 staining of muscle (A) and liver (B). Boxed regions are enlarged on the right. Phalloidin, actin filament staining. DAPI, nucleus staining.

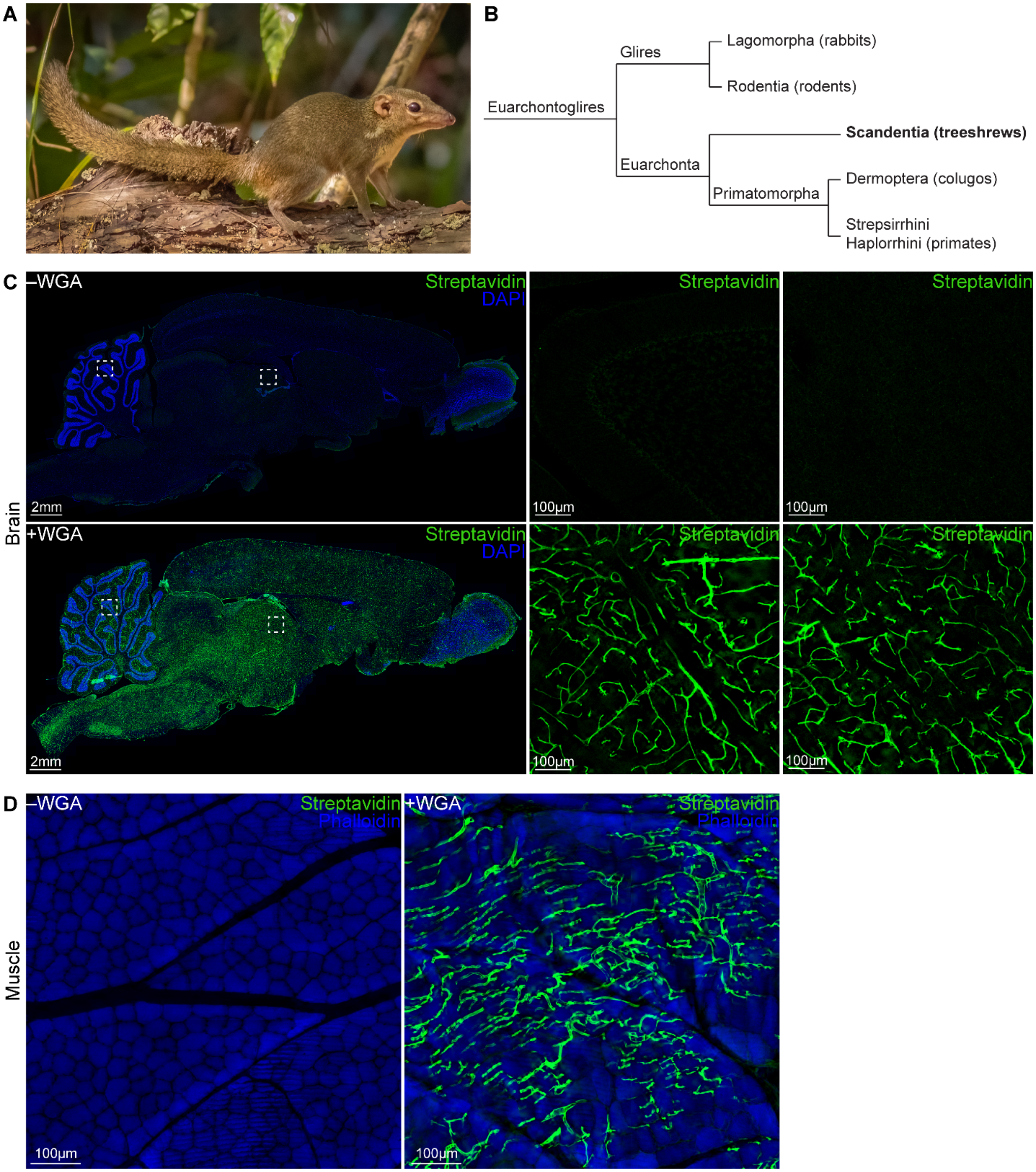

Fig. S2. *In vivo* biotinylation of the vasculature luminal surface in treeshrew.
(**A**) A northern treethrew (*Tupaia belangeri*) in its natural habitat. Photo from Mike Rose and iNaturalist, used under the CC BY-NC 4.0 license. (**B**) Taxonomy and phylogenetic tree of treeshrews (*17*). (**C and D**) Streptavidin staining of treeshrew brain (C) and muscle (D). Boxed regions are enlarged on the right. DAPI, nucleus staining. Phalloidin, actin filament staining. –WGA, WGA-HRP omitted. +WGA, WGA-HRP perfused.

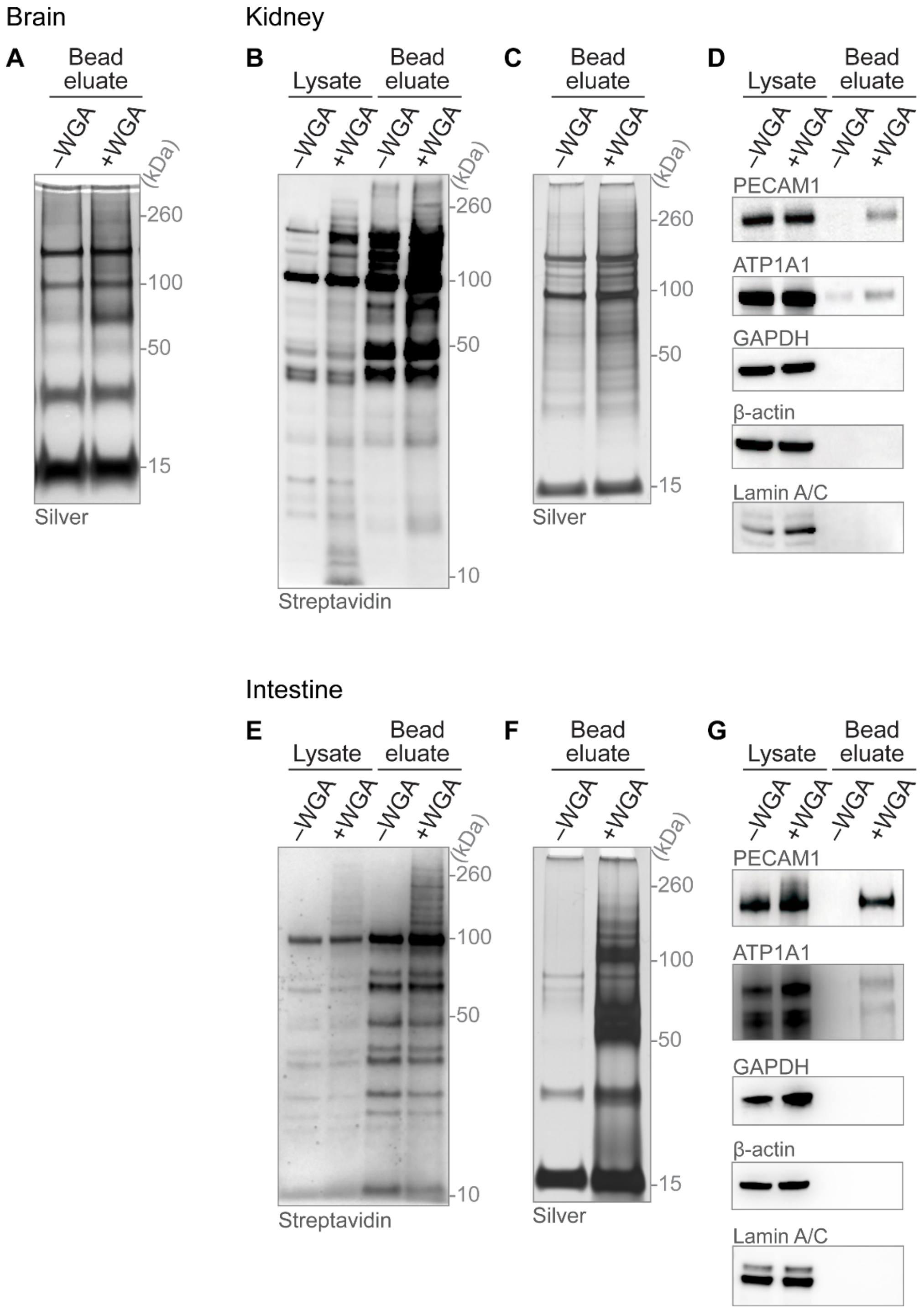

Fig. S3. Biochemical assays on brain, kidney, and intestine samples.
(**A**) Silver gel stain of post-enrichment bead eluates of brain samples. (**B to D**) Streptavidin blot (B), silver gel stain (C), and Western blots (D) of raw kidney lysates and post-enrichment bead eluates. (**E to G**) Streptavidin blot (E), silver gel stain (F), and Western blots (G) of raw intestine lysates and post-enrichment bead eluates. PECAM1, an endothelial cell marker. ATP1A1, a cell-surface Na^+/K^+ ATPase. GAPDH, β-actin, and lamin A/C, intracellular proteins. –WGA, WGA-HRP omitted. +WGA, WGA-HRP perfused.

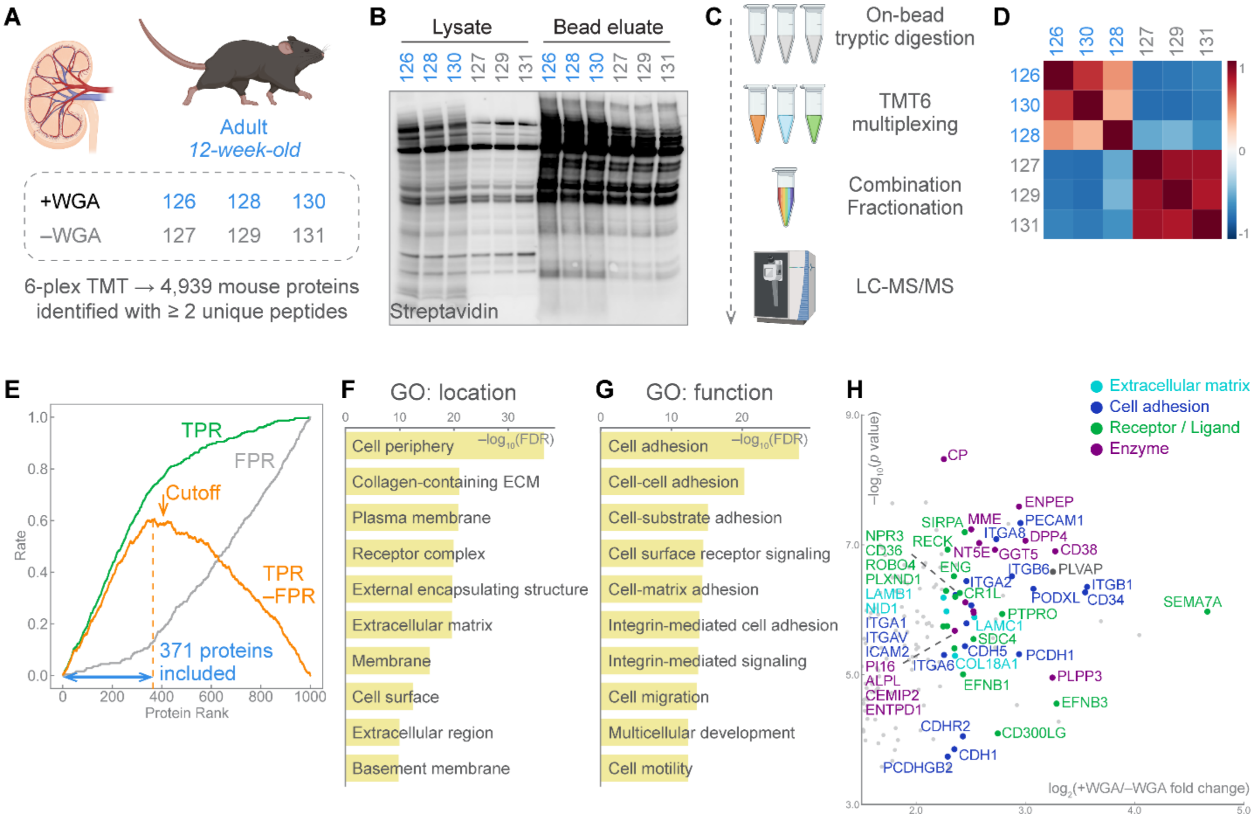

Fig. S4. Luminal surface proteome of the adult mouse kidney vasculature.
(**A**) Design and TMT tag usage of the 6-plex quantitative proteomic experiment, comprising three biotinylated replicates (blue) and three non-biotinylated controls (gray). A total of 4,939 mouse proteins were detected with two or more unique peptides (**table S5**). (**B**) Streptavidin blot of raw tissue lysates and post-enrichment bead eluates from kidney samples that were used for mass spectrometry analysis. (**C**) Workflow of sample preparation and mass spectrometry analysis. (**D**) Correlation of 6 TMT channels. (**E**) Plot of true positive rate (TPR, green), false positive rate (FPR, gray), and their difference (TPR–FPR, orange) for the proteomic data. The cutoff position (arrow) was set where the value of TPR–FPR was maximal. 371 proteins ranked above this cutoff position (to the left of the cutoff line on the plot) were retained (**table S6**). (**F and G**) Top ten gene ontology features of the post-cutoff 371 proteins: location (F) and function (G). FDR, false discovery rate. (**H**) Most enriched proteins at the luminal surface of the mouse kidney vasculature, including blood vessel markers CD34, CDH5, and PECAM1. Proteins are colored based on their molecular families: cyan, extracellular matrix; blue, adhesion molecule; green, receptor or ligand; purple, enzyme. +WGA, WGA-HRP perfused. –WGA, WGA-HRP omitted.

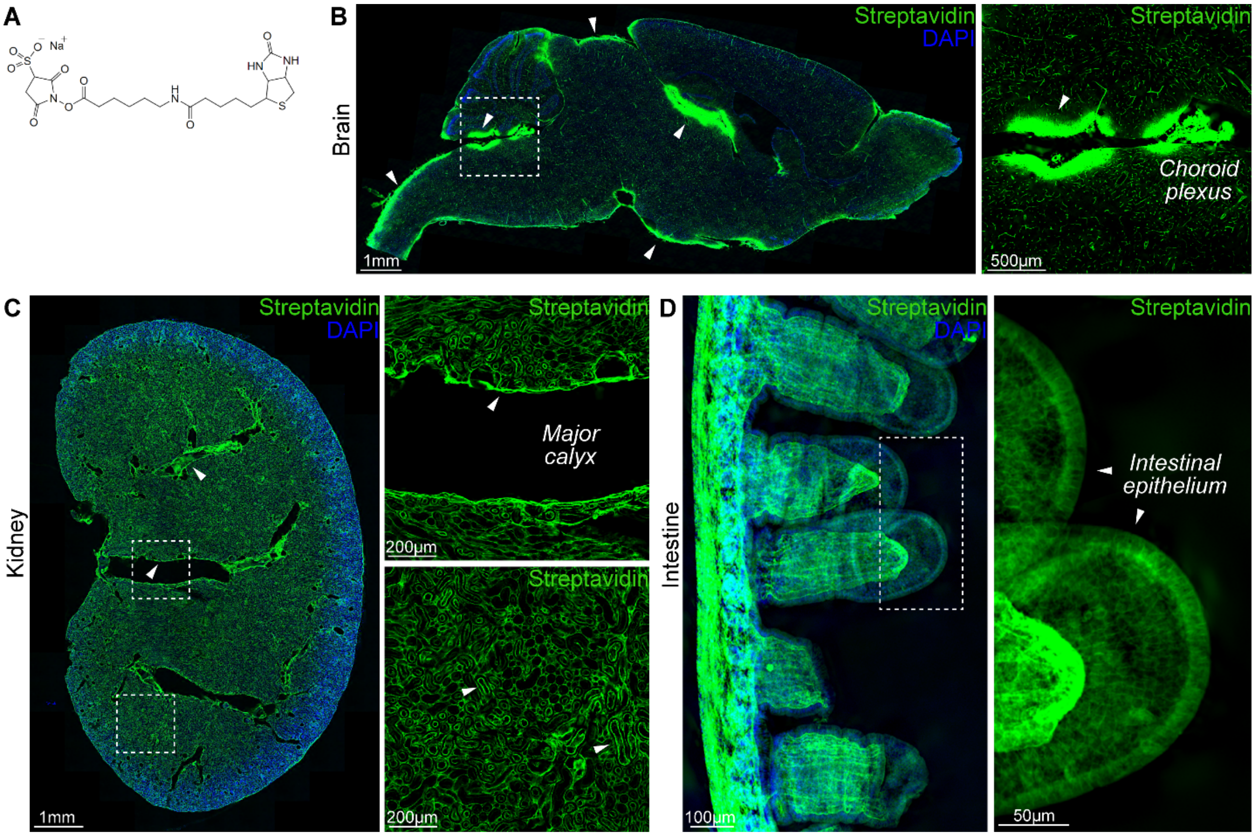

Fig. S5. Sulfo-NHS-LC-biotin perfusion labels non-vasculature compartments.
(**A**) Chemical structure of Sulfo-NHS-LC-biotin. (**B to D**) Streptavidin staining of brain (B), kidney (C), and intestine (D) after Sulfo-NHS-LC-biotin perfusion. Boxed regions are enlarged on the right. White arrowheads indicate non-vasculature compartments labelled by Sulfo-NHS-LC-biotin.

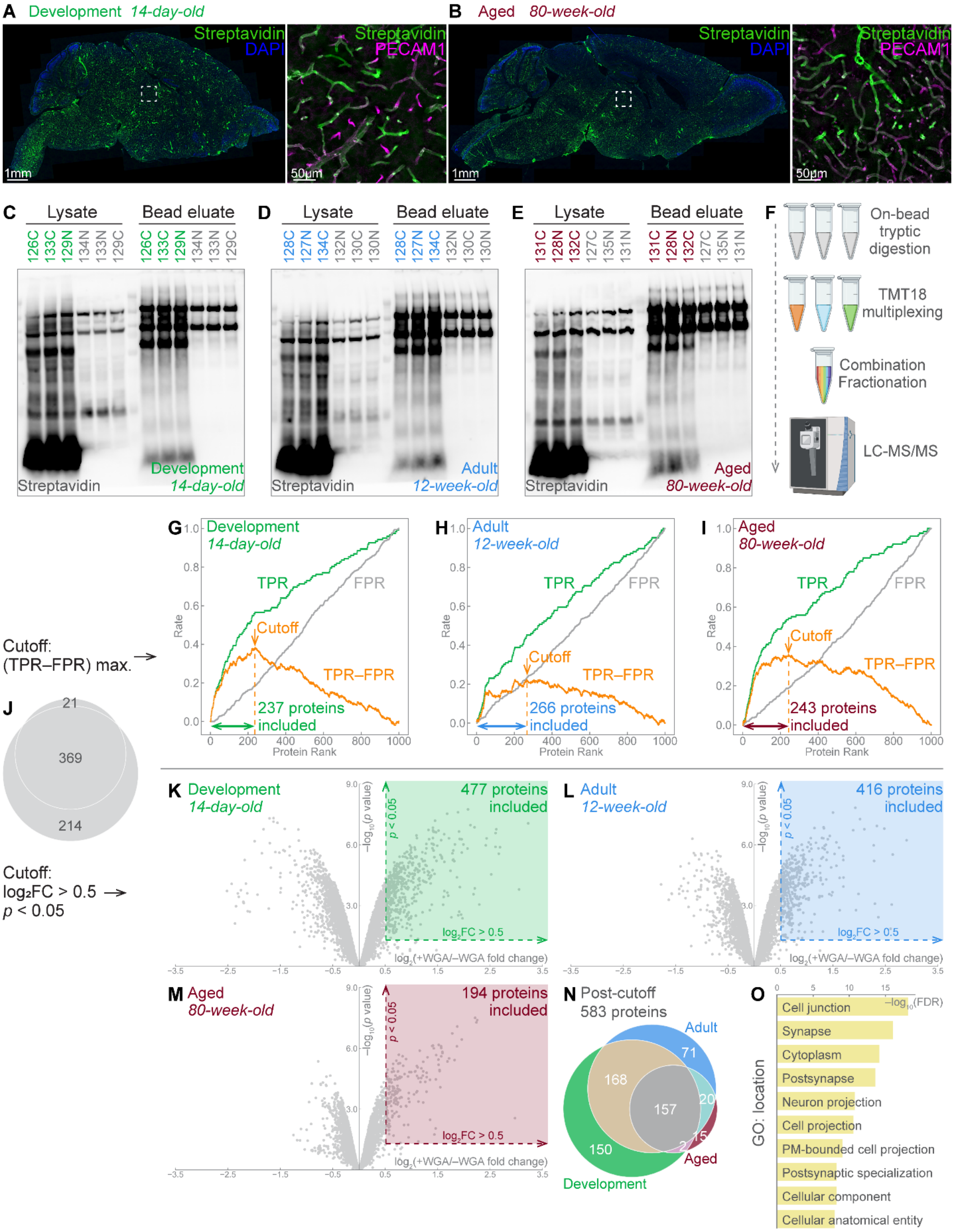

Fig. S6. Proteomic sample collection and data analysis.

(**A and B**) Streptavidin and anti-PECAM1 staining of neonatal (A) and aged (B) mouse brains after the biotinylation reaction. Boxed regions are enlarged on the right. DAPI, nucleus staining. (**C to E**) Streptavidin blots of raw brain lysates and post-enrichment bead eluates from development (C), adult (D), and aged (E) samples that were used for mass spectrometry analysis. (**F**) Workflow of sample preparation and mass spectrometry analysis. (**G to I**) Plots of true positive rate (TPR, green), false positive rate (FPR, gray), and their difference (TPR–FPR, orange) for the development (G), adult (H), and aged (I) proteomic data. For each stage, the cutoff position (arrow) was set where the value of TPR–FPR was maximal. Proteins ranked above this cutoff position (to the left of the cutoff line on the plot) were retained. (**J**) Venn diagram comparing the results of two different cutoff methods. (**K to M**) An alternative cutoff method retaining proteins with $\log_2$(+WGA/–WGA fold change) > 0.5 and $p < 0.05$ at each stage: development (K), adult (L), and aged (M). (**N**) Venn diagram of the post-cutoff proteome. (**O**) Top ten gene ontology location features of the post-cutoff 583 proteins. FDR, false discovery rate. +WGA, WGA-HRP perfused. –WGA, WGA-HRP omitted. FC, fold change.

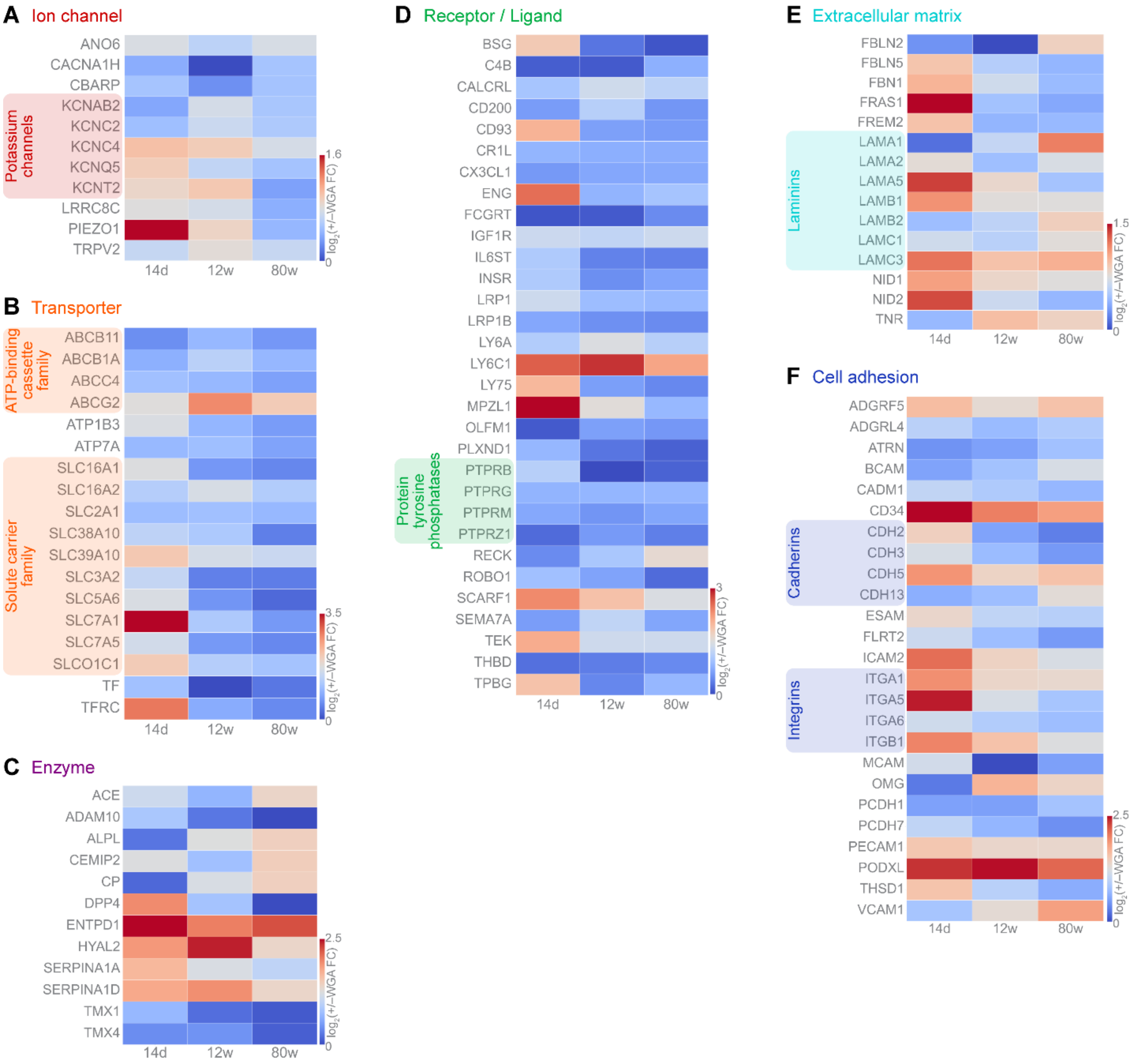

Fig. S7. Molecular families of luminal surface proteins and their expression dynamics across the lifespan.
Heatmaps showing the dynamics of individual proteins from development (14d, 14-day-old) through adulthood (12w, 12-week-old) to aging (80w, 80-week-old). Each protein is categorized based on its primary molecular family and most well-characterized function, although some proteins may align with multiple categories. +WGA, WGA-HRP perfused. –WGA, WGA-HRP omitted. FC, fold change.

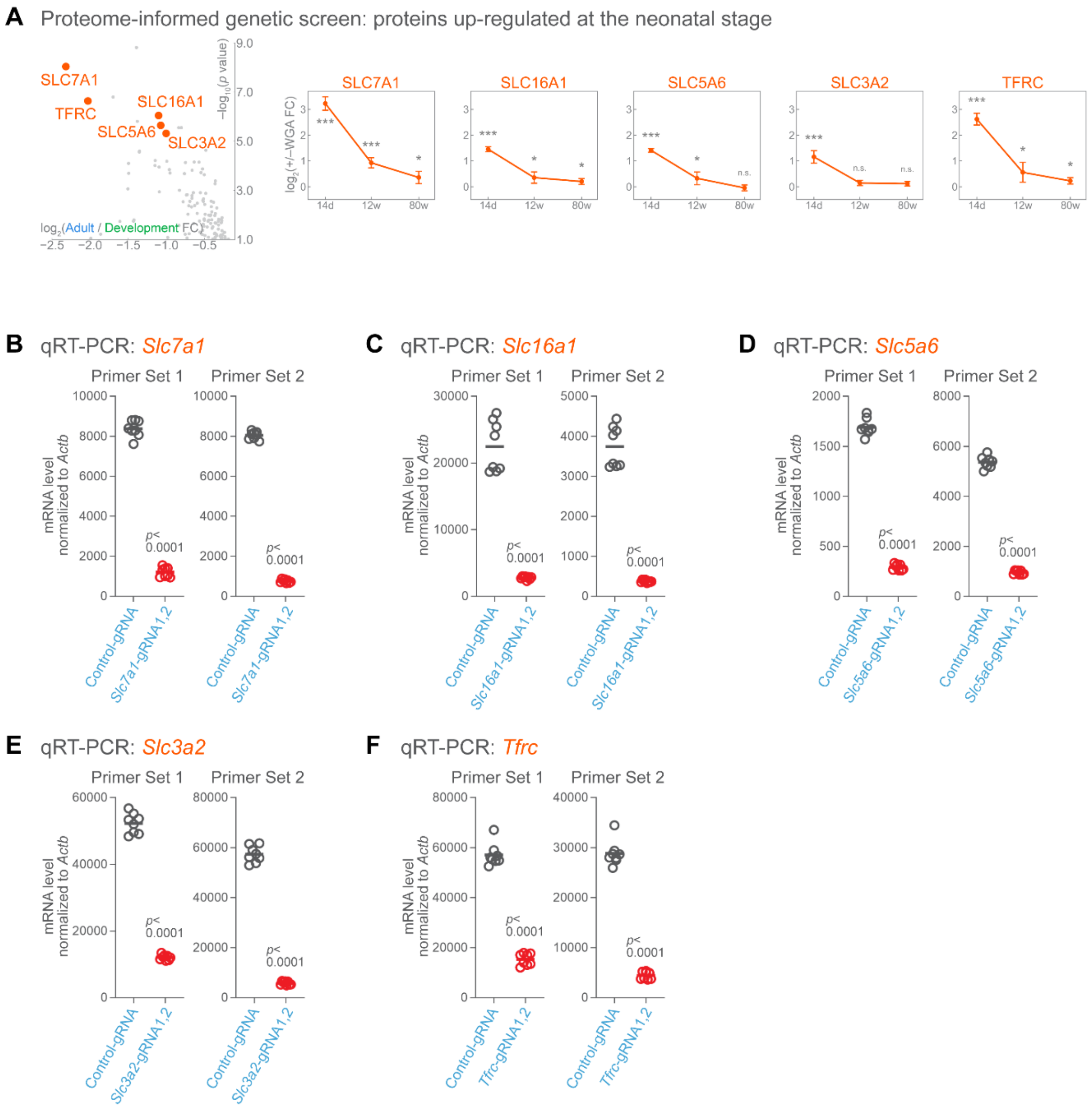

Fig. S8. *In vivo* genetic screen candidates and the validation of CRISPR/Cas9-mediated gene knockout.

(**A**) Using the viral-genetic strategy described in Fig. 4A, we knocked out five transport-related luminal surface proteins that are developmentally enriched, including four solute carrier transporters and a transferrin receptor. Their expression dynamics are plotted. Y axis, $\log_2$(+WGA/–WGA fold change). +WGA, WGA-HRP perfused. –WGA, WGA-HRP omitted. FC, fold change. 14d, 14-day-old. 12w, 12-week-old. 80w, 80-week-old. Error bar, standard deviation. Asterisk, p value associated with the +WGA/–WGA fold change to assess whether a protein is enriched at each stage. ***, $p < 0.001$. **, $p < 0.01$. *, $p < 0.05$. n.s., not significant. (**B to F**) Quantitative reverse transcription PCR (qRT-PCR) validation of CRISPR/Cas9-mediated gene knockout. For each target gene, two distinct primer pairs (sequences provided in the Materials and Methods) were used to quantify transcript amounts in RNA extracted from two mouse brains, with four PCR technical replicates per brain sample. mRNA expression was normalized to *Actb* (β-actin). Two-tailed t test was used to compare each knockout with its corresponding control.

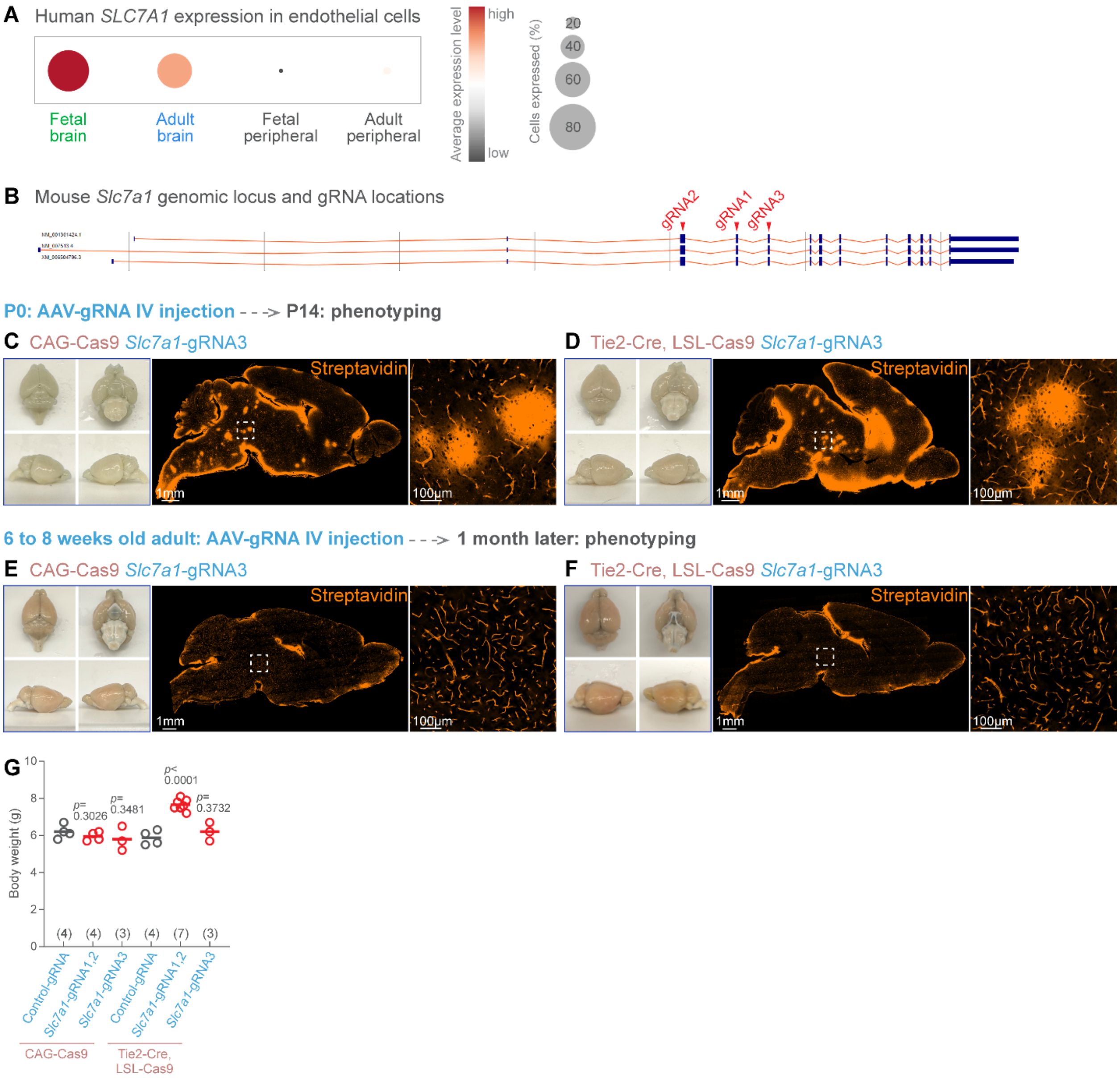

Fig. S9. Identification of SLC7A1 as a blood-brain barrier regulator in neonates.
(**A**) *SLC7A1* expression in human endothelial cells, revealed by RNA sequencing (*58*). (**B**) Mouse *Slc7a1* genomic locus and *Slc7a1*-targeting guide RNA locations. (**C and D**) *Slc7a1* knockout by a single guide RNA (*Slc7a1*-gRNA3) in either body-wide (C) or endothelial cell specific Cas9 (D) mice at the neonatal stage. Left, whole brain photos showing the Evans blue stain. Middle, streptavidin staining of the brain sagittal section for Sulfo-NHS-LC-biotin detection. Right, zoom-in of the boxed region. (**E and F**) *Slc7a1* knockout by a single guide RNA (*Slc7a1*-gRNA3) in young adult mice. E, body-wide Cas9. F, endothelial cell specific Cas9. (**G**) Body weights of control and *Slc7a1* knockout mice at postnatal day 14. Numbers in the parentheses show the total mouse number of each experimental condition. Two-tailed *t* test was used to compare each knockout with its corresponding control.

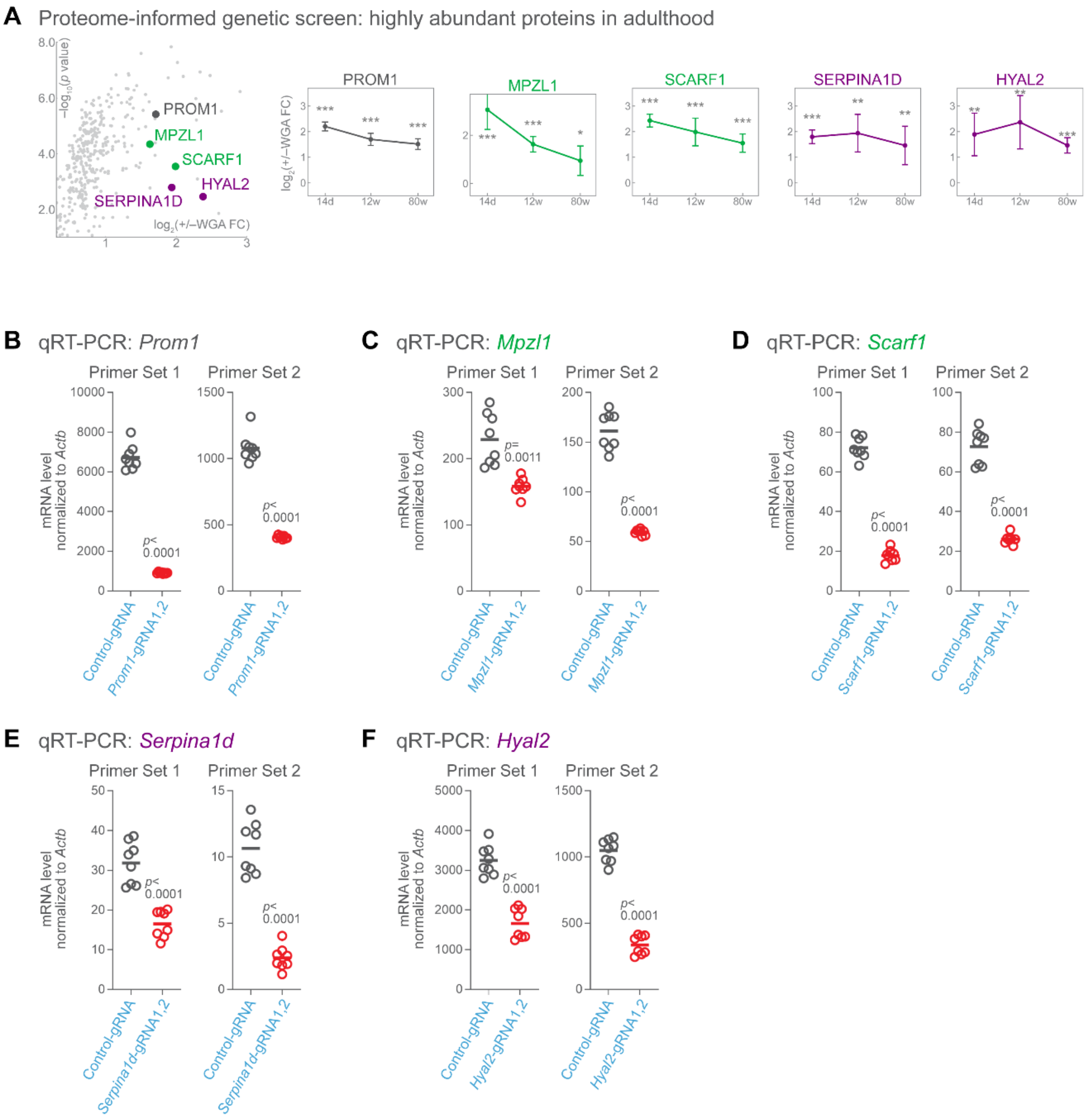

Fig. S10. *In vivo* genetic screen candidates and the validation of CRISPR/Cas9-mediated gene knockout.

(**A**) Using the viral-genetic strategy described in Fig. 4K, we knocked out five luminal surface proteins that are abundantly expressed in adulthood but barely studied previously. Their expression dynamics are plotted. Y axis, $\log_2$(+WGA/–WGA fold change). +WGA, WGA-HRP perfused. –WGA, WGA-HRP omitted. FC, fold change. 14d, 14-day-old. 12w, 12-week-old. 80w, 80-week-old. Error bar, standard deviation. Asterisk, p value associated with the +WGA/–WGA fold change to assess whether a protein is enriched at each stage. ***, $p < 0.001$. **, $p < 0.01$. *, $p < 0.05$. n.s., not significant. (**B to F**) Quantitative reverse transcription PCR (qRT-PCR) validation of CRISPR/Cas9-mediated gene knockout. For each target gene, two distinct primer pairs (sequences provided in the Materials and Methods) were used to quantify transcript amounts in RNA extracted from two mouse brains, with four PCR technical replicates per brain sample. mRNA expression was normalized to *Actb* (β-actin). Two-tailed t test was used to compare each knockout with its corresponding control.

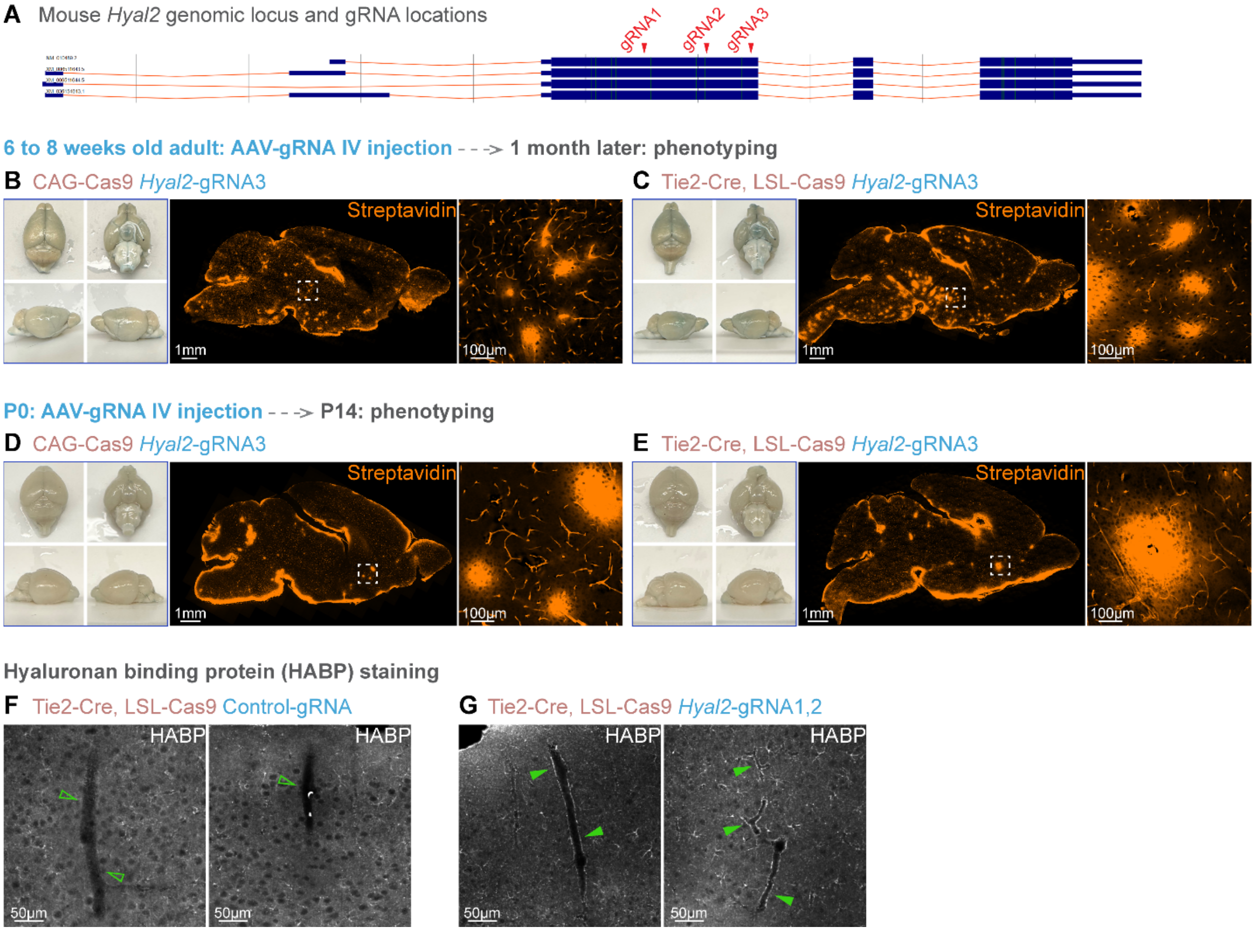

Fig. S11. Identification of HYAL2 as a blood-brain barrier regulator.
(**A**) Mouse *Hyal2* genomic locus and *Hyal2*-targeting guide RNA locations. (**B and C**) *Hyal2* knockout by a single guide RNA (*Hyal2*-gRNA3) in young adult mice expressing body-wide Cas9 (B) or endothelial cell specific Cas9 (C). Left, whole brain photos showing the Evans blue stain. Middle, streptavidin staining of the brain sagittal section for Sulfo-NHS-LC-biotin detection. Right, zoom-in of the boxed region. (**D and E**) *Hyal2* knockout by a single guide RNA (*Hyal2*-gRNA3) in either body-wide (D) or endothelial cell specific Cas9 (E) mice at the neonatal stage. (**F and G**) Hyaluronan binding protein (HABP) staining of control (F) and *Hyal2* knockout (G) brain sections. Empty arrowhead, absence of HABP staining along the blood vessel. Filled arrowhead, HABP signal lining the blood vessel.

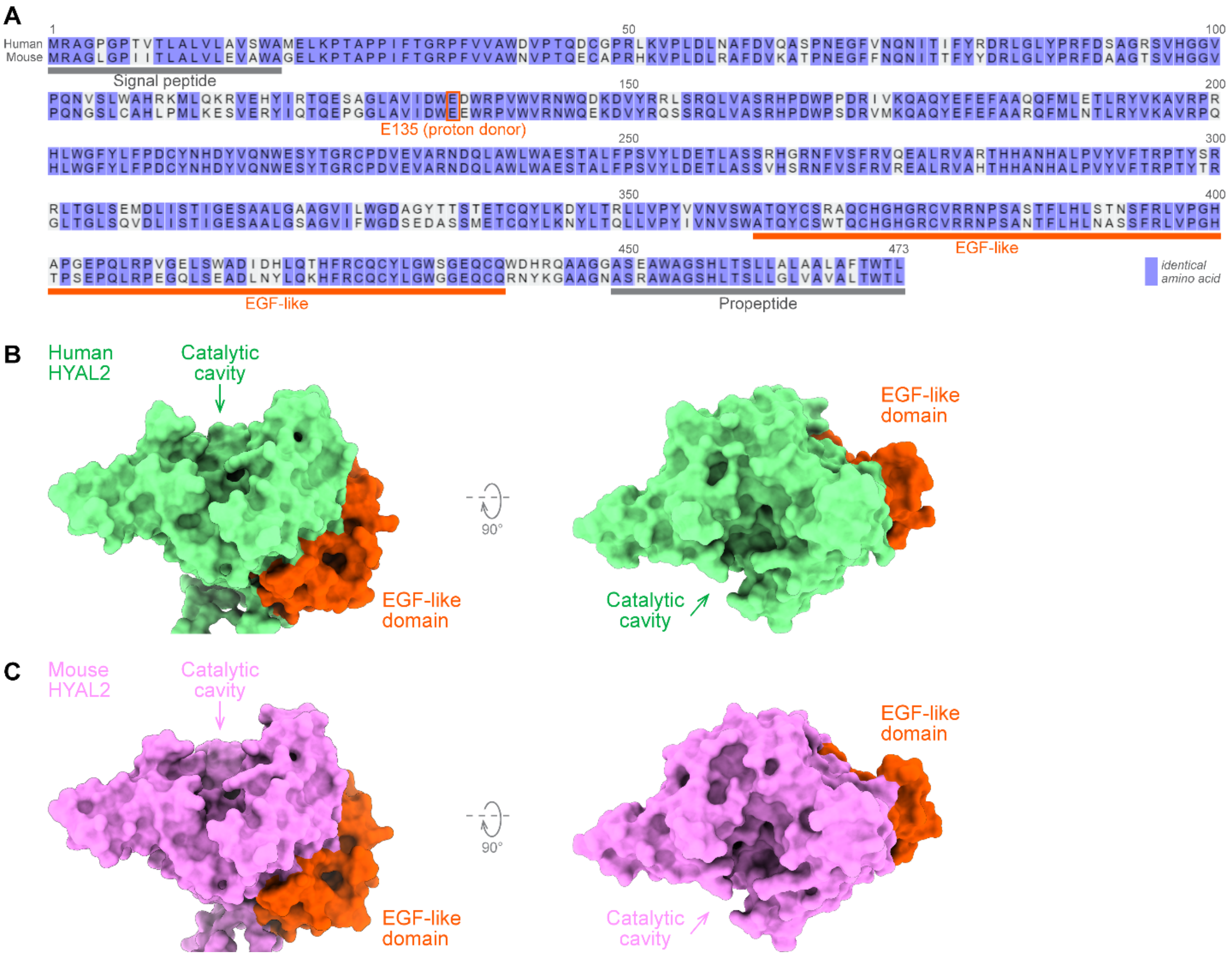

Fig. S12. Sequence alignment and structure analysis of human and mouse HYAL2.
(**A**) Amino acid sequence alignment of human and mouse HYAL2 proteins, with feature regions highlighted: signal peptide, proton donor glutamic acid (E135), EGF-like domain, and propeptide. Purple, identical amino acid. (**B and C**) Surface renderings of human and mouse HYAL2 structures showing their structural similarity and the location of the catalytic cavity and the EGF-like domain.

Table S1. Brain — all 4,528 mouse proteins detected with 2 or more unique peptides.
(**A**) TMT ratios. (**B**) Biotinylated-to-control ('+WGA' to '–WGA') fold changes at each age and their corresponding *p* values. 14d, 14-day-old. 12w, 12-week-old. 80w, 80-week-old.

Table S2. Brain — post-cutoff 390 proteins depicted in Fig. 2D.
(**A**) 237 proteins of the development stage (14d, 14-day-old). (**B**) 266 proteins of the adult stage (12w, 12-week-old). (**C**) 243 proteins of the aged stage (80w, 80-week-old). (**D**) Union of A–C: 390 proteins that passed cutoff at one or more stages. (**E**) Intersection of A–C: 120 proteins that passed cutoff at all three stages.

Table S3. Brain — post-cutoff 583 proteins depicted in fig. S6N.
(**A**) 477 proteins of the development stage (14d, 14-day-old). (**B**) 416 proteins of the adult stage (12w, 12-week-old). (**C**) 194 proteins of the aged stage (80w, 80-week-old). (**D**) Union of A–C: 583 proteins that passed cutoff at one or more stages. (**E**) Intersection of A–C: 157 proteins that passed cutoff at all three stages.

Table S4. Brain — expression dynamics of the post-cutoff 390 proteins depicted in Fig. 2D.
Expression fold changes between two ages and their corresponding *p* values. 14d, 14-day-old. 12w, 12-week-old. 80w, 80-week-old.

Table S5. Kidney — all 4,939 mouse proteins detected with 2 or more unique peptides.
(**A**) TMT ratios. (**B**) Biotinylated-to-control ('+WGA' to '–WGA') fold changes and their corresponding *p* values.

Table S6. Kidney — post-cutoff 371 proteins depicted in fig. S4E.
Biotinylated-to-control ('+WGA' to '–WGA') fold changes and their corresponding *p* values of the post-cutoff 371 proteins.
